# An *in vivo* Cell-Based Delivery Platform for Zinc Finger Artificial Transcription Factors in Pre-clinical Animal Models

**DOI:** 10.3389/fnmol.2021.789913

**Published:** 2022-01-27

**Authors:** Peter Deng, Julian A. N. M. Halmai, Ulrika Beitnere, David Cameron, Michele L. Martinez, Charles C. Lee, Jennifer J. Waldo, Krista Thongphanh, Anna Adhikari, Nycole Copping, Stela P. Petkova, Ruth D. Lee, Samantha Lock, Miranda Palomares, Henriette O’Geen, Jasmine Carter, Casiana E. Gonzalez, Fiona K. B. Buchanan, Johnathan D. Anderson, Fernando A. Fierro, Jan A. Nolta, Alice F. Tarantal, Jill L. Silverman, David J. Segal, Kyle D. Fink

**Affiliations:** ^1^Department of Neurology, University of California Davis School of Medicine, Sacramento, CA, United States; ^2^Stem Cell Program and Gene Therapy Center, University of California, Davis, Sacramento, CA, United States; ^3^Department of Biochemistry and Molecular Medicine, Genome Center, University of California, Davis, Davis, CA, United States; ^4^Department of Psychiatry and Behavioral Sciences, MIND Institute, University of California Davis School of Medicine, Sacramento, CA, United States; ^5^Departments of Pediatrics and Cell Biology and Human Anatomy, School of Medicine, Gene Therapy Center, and California National Primate Research Center, University of California, Davis, Davis, CA, United States; ^6^Department of Otolaryngology, University of California, Davis, Davis, CA, United States

**Keywords:** zinc finger, Angelman Syndrome (AS), mesenchymal stem/stromal cell, artificial transcription factor (ATF), cell-based delivery

## Abstract

Zinc finger (ZF), transcription activator-like effectors (TALE), and CRISPR/Cas9 therapies to regulate gene expression are becoming viable strategies to treat genetic disorders, although effective *in vivo* delivery systems for these proteins remain a major translational hurdle. We describe the use of a mesenchymal stem/stromal cell (MSC)-based delivery system for the secretion of a ZF protein (ZF-MSC) in transgenic mouse models and young rhesus monkeys. Secreted ZF protein from mouse ZF-MSC was detectable within the hippocampus 1 week following intracranial or cisterna magna (CM) injection. Secreted ZF activated the imprinted paternal *Ube3a* in a transgenic reporter mouse and ameliorated motor deficits in a *Ube3a* deletion Angelman Syndrome (AS) mouse. Intrathecally administered autologous rhesus MSCs were well-tolerated for 3 weeks following administration and secreted ZF protein was detectable within the cerebrospinal fluid (CSF), midbrain, and spinal cord. This approach is less invasive when compared to direct intracranial injection which requires a surgical procedure.

## Introduction

Historically, engineered stem cell therapies have been utilized as a gene replacement and cross-correction approach by producing proteins at supraphysiologic levels *in vivo* (Kohn et al., 1995, 1998, 2021; Visigalli et al., 2010; Pollock et al., 2016; Adhikari et al., 2019, 2021; Beegle et al., 2020). Mesenchymal stem/stromal cells (MSCs) are a particularly favorable cell type as they are easily obtained, expanded, and can be readily manipulated *ex vivo* (Meyerrose et al., 2010; Damasceno et al., 2020). Additionally, MSCs do not permanently engraft into the host tissue (Kean et al., 2013), do not induce immune responses through the secretion of immunomodulatory cytokines, and demonstrate a clinically favorable safety profile (Thompson et al., 2020). These unique characteristics of MSCs make them an attractive potential delivery vehicle for gene modifying artificial transcription factors (ATF) such as zinc finger (ZF), transcription activator-like effectors (TALE), and CRISPR/Cas9 (Russ and Lampel, 2005; Thakore et al., 2015; Bailus et al., 2016; Fink et al., 2016; Halmai et al., 2020). The identification of an effective delivery system remains a challenge in the gene therapy field as these gene modifying approaches become increasingly sophisticated for the treatment of genetic disorders. Direct administration of purified protein (Bailus et al., 2016; Perdigão et al., 2020), lipid nanoparticle (Wang et al., 2016; Cheng et al., 2020), and viral-mediated approaches such as adeno-associated virus (Mendell et al., 2017; Russell et al., 2017) have been explored as putative delivery systems for ATF *in vivo*; however, a cell-based system such as MSCs has not been reported to date.

The most common cause for Angelman Syndrome (AS) is the loss of *UBE3A* in mature neurons due to a deletion of the maternal *UBE3A* allele and silencing of the paternal allele *via* a long non-coding RNA (Kishino et al., 1997; Chamberlain and Lalande, 2010). We previously described the use of a systemically administered, purified ZF-KRAB protein that achieved brain-wide distribution and activation of paternal *Ube3a* in a mouse model of AS by targeting ZF to the *Snurf/Snrpn* locus, a putative promoter for the *Ube3a-* antisense transcript (Landers et al., 2004; Bailus et al., 2016). Here, we evaluate engineered MSCs as a novel delivery platform for ZF gene modifiers into the central nervous system (CNS) of pre-clinical mouse models and juvenile rhesus monkeys. We observed that ZF secreting MSCs can secrete full-length, biologically active protein *in vitro*. Secreted ZF functionality was evaluated in two mouse models of AS: a paternal *Ube3a* reporter mouse (*Ube3a^+/yfp^*) and a maternal *Ube3a* deletion model (*Ube3a^mat–/pat+^*). Both intracranial and cisterna magna (CM) injection of ZF-MSC resulted in protein uptake in neuronal tissue and activation of a paternal *Ube3a* reporter across multiple brain regions in mice 3 weeks following injection. ZF-MSC treatment in the *Ube3a^mat–/pat+^* AS mouse model also resulted in attenuated motor deficits up to 29 days post-injection. Lastly, studies were conducted in young rhesus monkeys to evaluate the translational feasibility, safety, and tolerability of ZF-MSCs in a pre-clinical animal model with close similarities to human anatomy and physiology. ZF-MSCs were well tolerated following intrathecal (IT) administration. Secreted ZF protein was detected in the cerebrospinal fluid (CSF), midbrain, and spinal cord 3 weeks following administration of ZF-MSCs. Overall, this study demonstrates the functionality of an MSC-based secretion platform for biologically active ATF *in vivo*.

## Materials and Methods

### Vector Design and Lentiviral Packaging

IgK-Leader sequence with TATp was Gibson cloned into the original *pMAL-TatP-mCherry-HA-NLS-ZF00-KRAB via Sal*I and *Asc*I. The transgene was excised from pMAL backbone *via Sal*I (NEB, Ipswich, MA, United States) and *Pst*I (NEB), followed by blunting with DNA Polymerase I (NEB) cloned into our pCCLc-MNDU3-X2-WPRE vector *via Sma*I (NEB) restriction site. Clones were subsequently Sanger sequenced confirmed (Quintara Biosciences, South San Francisco, CA, United States). Lentivirus was prepared accordingly to previously published protocols (Kalomoiris et al., 2012). Briefly, lentivirus was generated by complexing 25 μg of transgene vector, 25 μg of delta 8.9, and 5 μg were VSVG with 145 μg of PEI in 3 ml serum-free DMEM-HG (Thermo Fisher Scientific, Waltham, MA, United States, Catalog #11965118) for 20 min prior to application to 2.5 × 10^6^ Lenti-X 293 cells (Takara Bio, Kusatsu, Japan, Catalog #632180). Twenty-four hours following transfection, cells were switched to serum-free Ultraculture (Lonza, Basel, Switzerland, Catalog #BP12-725F) for 48 h. Viral harvest was performed by transferring the supernatant of transfected Lenti-X 293 cells into 50 ml conical tubes, centrifuged at 500 RPM for 5 min to pellet cellular debris, and virus was isolated in a 100 kDa Centricon (MilliporeSigma, Burlington, MA, United States, Catalog #UFC710008).

### Cell Isolation and Culturing

#### Mouse Bone-Marrow Mesenchymal Stem/Stromal Cell

The extraction protocol for obtaining MSCs was adapted from previously published procedures (Rossignol et al., 2011). Briefly, bone marrow was aspirated from the fibula of adult (6–8-week-old) C57BL/6 mice (Jackson Laboratory, Bar Harbor, ME, United States, Catalog # C57BL/6J) using a 25-gauge needle and attached 3 ml syringe. The cells were then suspended in 10 ml MSC medium (alpha modified Eagle’s medium, Sigma, St Louis, MO, United States, Catalog # D5030) with 20% fetal bovine serum (FBS) (Invitrogen, Carlsbad, CA, United States, Catalog #16000044) and 5 mg/ml streptomycin and 5 UI/ml penicillin (Sigma, Catalog #85886). Following incubation for 48 h at 37°C, 5% CO_2_, and 20% O_2_ the medium was replaced with fresh MSC medium to remove non-adherent cells. MSCs were passaged, expanded, and cryopreserved over passages 3–8. Passage 5 MSCs were transduced with competent lentivirus at MOI 100 and confirmed for transduction *via* fluorescent microscopy and sequencing. Vector copy number/cell was determined as WPRE/2GAPDH and verified as ∼2–5 vector transgene copies per cell. Primers/probes were designed based on published sequences and are as follows: moGAPDH- 5′-ACC ACG AGA AAT ATG ACA ACT-3′, 5′-CCC ACT GCC TAC ATA CCA TGA-3′, and 5′/56-FAM/TCA GCA ATG CAT CCT GCA CCA CC/36-TAMSp/3′. WPRE—5′-TTA CGC TAT GTG GAT ACG CTG-3′, 5′-TCA TAA AGA GAC AGC AAC CAG G-3′, and 5′/56-FAM/AGG AGA AAA TGA AAG CCA TAC GGG AAG C/36-TAMSp/3′. Quantitative PCR was performed using TaqMan Universal PCR Master Mix, no AmpErase UNG (Life Technologies, Catalog# 4304437), and the indicated primers/probes under the following reaction conditions on an Applied Biosystems StepOne Plus (Thermo Fisher Scientific, Waltham, MA, United States, Catalog #4376600): Stage 1—50°C for 2 min, Stage 2—95°C for 10 min, Stage 3—40 cycles of 95°C for 15 s and 60°C for 1 min, and Stage 4 (dissociation)—95°C for 15 s, 60°C for 1 min, 95°C for 15 s, and 60°C for 15 s. Quantification was based on standard curves of plasmid DNA.

#### Mesenchymal Stem/Stromal Cell Characterization

For characterization for MSC by flow cytometry, cells were lifted by TrypLE and resuspended in PBS. Cells were then incubated with the respective conjugated antibodies individually (all diluted 1:100 in PBS, Supplementary Table 1) for 30 min at 4°C. For CD31, cells were washed once with PBS and then stained for additional 15 min with an PE-conjugated anti-rat IgG antibody. Then, all samples were washed once with 1 ml of PBS, centrifuged for 300 × *g* for 5 min and resuspended in PBS prior to analysis using a Attune flow cytometer. Negative controls (conjugated isotype antibodies and unlabeled cells) were used for gating.

#### Secreted Zinc Finger Protein Confirmation

Transduced MSCs were washed twice with PBS and incubated with OptiMem (Thermo Fisher Scientific, Waltham, MA, United States, Catalog #31985070) for 48 h before being concentrated in a 10-kDa Amicon ^®^ Ultra-15 Centrifugal Filter Unit (MilliporeSigma, Burlington, MA, United States, Catalog #UFC9030). This conditioned media was probed for presence of secreted protein with ms-HA (1:1,000, BioLegend, San Diego, CA, United States, Catalog #16B12) and Rb anti-β-Tubulin (1:2,000, Thermo Fisher Scientific, Waltham, MA, United States, Catalog #AA10).

#### Transwell Experiments

Neuro2As (ATCC, Manassas, VA, United States, Catalog #CCL-131) were cultured in DMEM-HG (Thermo Fisher Scientific, Waltham, MA, United States, Catalog #11965092), 10% FBS, and 1% L-Glutamine. 5 × 10^4^ Neuro2As were seeded per well in 12-well Transwell plates (Corning, Corning, NY, United States, Catalog #3401) and 2.5 × 10^4^ were seeded in Transwell baskets. Cells were equilibrated in their respective media for 24-h prior to co-incubation in reduced-serum Opti-Mem (Thermo Fisher Scientific, Waltham, MA, United States, Catalog #51985091) for longitudinal qPCR experiments. Purified ZF-P was provided by Dr. David Segal and spiked into Neuro2A cells at a dose of 100 μg/well. RNA was isolated using the Direct-Zol RNA Isolation Kit (Zymo Research, San Diego, CA, United States, Catalog #R2053). cDNA synthesis was performed using the RevertAid Random Hexamer Kit (Thermo Fisher Scientific, Waltham, MA, United States, Catalog #K1622). Ten nanograms of cDNA was probed with *Snrpn* primers (Snrpn-F 5′-TGCTACGTGGGGAGAACTTG-3′, Snrpn-R 5′-CCTGGGGAATAGGTACACCTG-3′) and *Gapdh* primers (Gapdh-F5′-TGACCACAGTCCATGCCATC-3′, Gapdh-R GACGGACACATTGGGGGTAG) with PowerUp SYBR Green (Thermo Fisher Scientific, Waltham, MA, United States, Catalog #A25741) in the Applied Biosystems StepOne Plus (Thermo Fisher Scientific, Waltham, MA, United States, Catalog #4376600). Data presented as 2^–ΔΔCt^ were normalized to the NT-MSC control group.

#### Primary Cortical Neuron Isolation and Culture

Primary cell cultures of cortical neurons were prepared from E15.5 *Ube3a^+/yfp^* prenatal mice. Brains were dissected and cortices isolated in ice-cold PBS (100 units/mL penicillin-streptomycin). Cortical tissue was dissociated by enzymatic digestion [50 units papain (Worthington, Catalog # LK003172), 250 units DNase (Sigma, Catalog #D4513) 1 mM L-cysteine, 0.5 mM EDTA (Sigma, Catalog #P0899) in HBSS] and mechanical trituration. Cells were filtered by density gradient centrifugation [HBSS, ovomucoid protease inhibitor (Worthington, Catalog #KL003182) with bovine serum albumin, BSA], counted, and plated on poly-D-lysine (Sigma, Catalog #A3890401) coated 12-well plates at 1 × 10^6^ cells per well. Cultures were grown in Neurobasal medium (Invitrogen, Catalog #21103-049) with 1× B-27 Supplement (Invitrogen) and 2 mM L-glutamine (Invitrogen, Catalog #25030-081) at 37°C and 5% CO_2_. At the first media change (72 h), 1 μM cytosine β-D-arabinofuranoside hydrochloride (Sigma, Catalog #C66415) was added to prevent replication of non-neuronal cells.

### Immunocytochemistry

#### Mesenchymal Stem/Stromal Cell

Transduced MSCs were washed with PBS and fixed using 10% formalin fixation for 10 min on ice. MSCs were blocked in 10% SEABLOCK (Thermo Fisher Scientific, Catalog #37527) + PBS + 0.1% Triton X100 (VWR, Catalog #0694-1L) for 1 h prior to incubation with primary antibody 1:500 rb-mCherry (abcam, Boston, MA, United States, Catalog #ab167453) and 1:1,000 ms-Nestin (abcam, Catalog# 22035) for 1.5 h. MSCs were rinsed 3× with ice cold PBST and incubated with AlexaFluor secondary antibody 1:1,000 goat rb-594 (Thermo Fisher Scientific, Catalog #A32740) and 1:1,000 goat ms-488 (Thermo Fisher Scientific, Catalog #A28175) for 1.5 h and then with 1:1,000 Hoechst (Cell Signaling, Catalog #4082) for 10 min. MSCs were then rinsed 2× with PBST before storing in PBS. Cells were imaged on a Nikon Eclipse Ti-U inverted research microscope (Nikon, Melville, NY, United States).

#### Primary Cortical Neurons

Treated neurons were washed with PBS and fixed using 10% formalin fixation for 10 min on ice. Neurons were blocked in 10% SEABLOCK (Thermo Fisher Scientific, Catalog #37527) + PBS + 0.1% Triton X100 (VWR, Catalog #0694-1L) prior to incubation with primary antibody 1:500 rb-GFP (1:1,000, Novus Biologics, Centennial, CO, United States, Catalog #NB600-308) for 1 h. Neurons were rinsed 3× with ice cold PBST and incubated with secondary antibody 1:1,000 goat rb-488 (Alexa Fluor, Catalog #A32731) for 1 h. Neurons were then rinsed 2× with PBST and imaged on a Nikon Eclipse Ti-U inverted research microscope (Nikon, Melville, NY, United States).

### Mice

All experimental procedures were performed in accordance with the National Institutes of Health Guide for Care and Use of Laboratory Animals and were approved by the Institutional Animal Care and Use Committees (IACUC) of the University of California, Davis. A total of 81 mice were used for the following studies. Eleven FVB mice were used for initial MSC secretion characterization studies (7F, 4M) (Figure 3). Three *Ube3^matYfp/pat+^* were used as positive controls (2F, 1M) (Figures 3–5). Twenty-one *Ube3a^+/yfp^* mice were used for 1-week co-localization studies (4F, 5M) and 3-week molecular studies (6F, 6M) (Figures 3–5). Forty-six *Ube3a^mat–/pat+^* were used for behavioral studies (21F, 25M) (Figure 6). Experimenters were blinded to treatment groups and genotypes until final data analysis was performed. All studies were sex balanced when possible.

**FIGURE 1:**
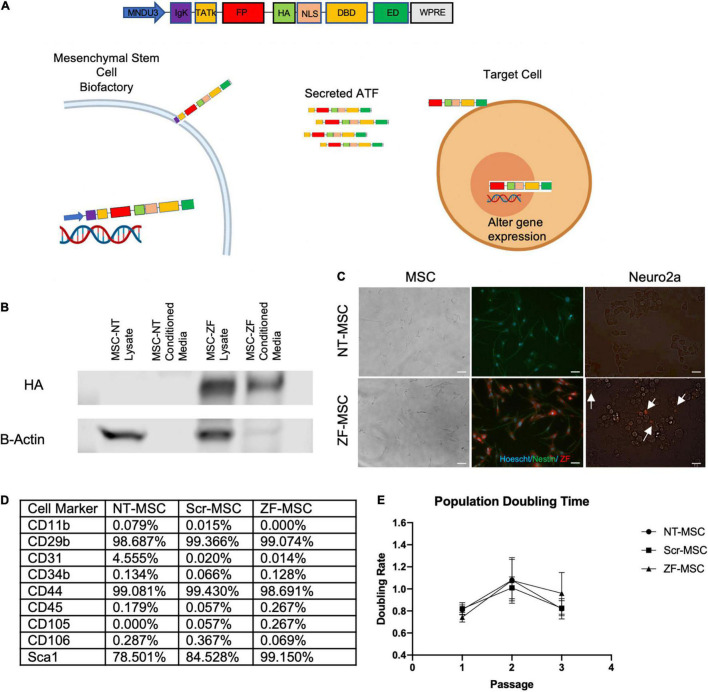
Engineered MSC can function as biofactories for zinc finger (ZF). **(A)** Schematic of design of secreted protein. Injected MSC biofactories secrete ZFs *via* the IgK Leader Signal Peptide into the extracellular space whereby these proteins are taken up by neighboring cells by the TATk element. ZFs are trafficked into the nucleus of target cells *via* NLS-tagged transport to alter target cell gene expression. **(B)** Western blot depicting presence of HA-tagged secreted ZF in conditioned media of MSC 48-h following serum starvation. **(C)** Left Panel: Fluorescent microscopy depicting Hoechst-labeled MSCs (blue) transduced to express ZF (red). Right Panel: Fluorescent microscopy depicting co-localization of ZF (red) on recipient Neuro2As following a 24-h incubation with conditioned media from ZF-MSCs. White arrows indicate Neuro2A that co-localize with ZF. Scale bar = 100 μm. **(D)** Table summary of FACS detailing the enrichment of isolated and transduced MSCs for typical MSC markers and negative for markers of cells of hemopoietic origin, related to Supplementary Figures 2, 3. **(E)** MSCs were passaged every 2 days and cell counts were recorded prior to plating. Transduction of MSCs to secrete either a Scr ZF or ZF did not demonstrate altered population doubling times compared to an isogenic NT-MSC. Error bars indicate ± standard error of the mean. Dots indicate average mean of four biological replicates. Two-Way ANOVA followed by a Tukey’s HSD. MNDU3, endogenous promoter; IgK, secretion peptide; TATk, furin-resistant TAT peptide; FPR, fluorescent protein reporter; HA, hemagglutinin epitope; NLS, nuclear localization signal; DBD, DNA-binding domain; ED, effector domain; WPRE, woodchuck hepatitis virus post-transcriptional element; ZF-MSC, MSCs that have been transduced to secrete ZF; NT-MSC, non-transduced MSC.

**FIGURE 2:**
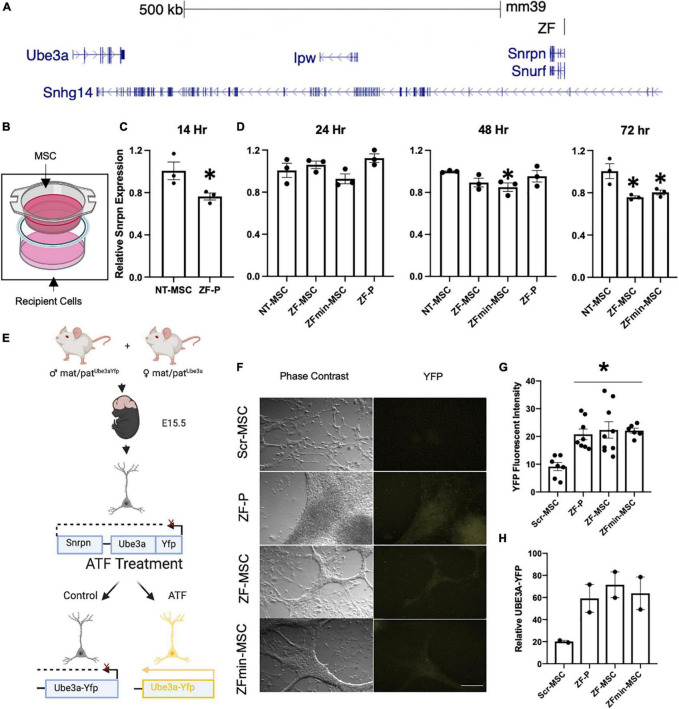
Secreted ZF protein demonstrates transcriptional effect in genes known in angelman syndrome. **(A)** Schematic of the ZF Binding to the putative promoter of the long non-coding RNA in the imprinted paternal *Ube3a* allele. **(B)** Schematic of transwell experiment, ZF-MSCs are seeded in the upper Transwell basket while recipient Neuro2As are seeded in the bottom well. **(C,D)** The efficacy of secreted ZF from MSC to repress *Snrpn* was assessed longitudinally in Neuro2a cells as measured by RT-qPCR. Each dot is representative of independent well. *n* = 3 independent wells per group. **(C)** An observable reduction of *Snrpn* expression was observed following a 14 h incubation with 100 μg purified ZF-P as a positive control for *Snrpn*, Student *T*-test, *p < 0.05 to NT-MSC repression. **(D)** An amelioration of ZF-P repression of *Snrpn* is observed by 24 h whereas ZF-MSC and ZFmin-MSC co-incubated groups demonstrate an observable decline of *Snrpn* expression over 48 h, [*F*_(3, 8)_ = 2.738] *p < 0.05 to NT-MSC, to 72 h, [*F*_(2, 6)_ = 9.225], *p < 0.05 to NT-MSC. **(E)** Schematic of experimental design for screening ZF in primary cortical neurons derived from E15.5 *Ube3a*^+/yfp^ pups. Successful interference of long non-coding RNA transcription activates paternally silent *Ube3a^yfp^*. **(F)** Immunocytochemistry panel of representative *Ube3a*^+/yfp^ primary cortical neurons shows activation of UBE3A-YFP (yellow) following incubation 24 h incubation with 250 μg of ZF-P, ZF-MSC CM, or ZFmin-MSCCM. Scale bar = 100 μm. **(G)** Quantification of YFP mean fluorescent intensity from **H** showed a significant two-fold increase in YFP fluorescent intensity in immune-labeled *Ube3a*^+/yfp^ primary cortical neurons. One-Way ANOVA [*F*_(3, 26)_ = 7.955] followed by Fisher’s LSD, *p < 0.05 from NT-MSC. Each dot is representative of an independent well containing an isolated cortex from a *Ube3a*^+/yfp^ pup. *n* = 7–9 wells per group. **(H)** Observable increases of UBE3A-YFP were visualized in western blot analysis of treated primary cortical neurons. Each dot is representative of an independent well containing an isolated cortex from a *Ube3a*^+/yfp^ pup. *n* = 2 wells per group. Yfp, yellow fluorescent protein. ZF-P, purified ZF protein. Error bars indicate ± standard error of the mean.

**FIGURE 3:**
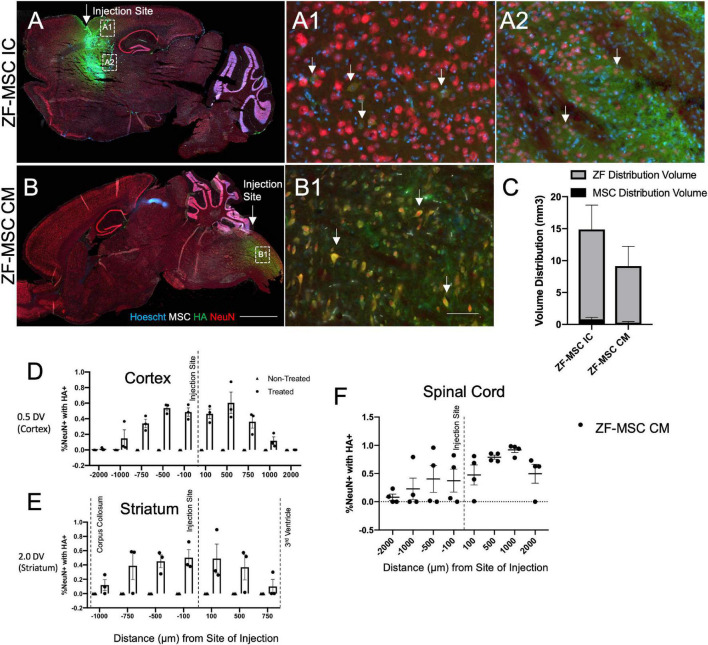
Secreted zinc finger (ZF) diffuse in a gradient fashion from site of injection. **(A)** Intracranial injection of ZF-MSC into the striatum resulted in an observable ZF (red) distribution. Uptake of ZF is observable in mature NeuN+ (red) neurons in the cortex **(A1)** and the striatum **(A2)**. White arrows indicate ZF+/NeuN+ cells. **(B)** CM injection of ZF-MSC demonstrated observable ZF distribution and uptake of protein NeuN+ neurons in the spinal cord **(B1)**. White arrows indicate ZF+/NeuN+ cells. **(C)** Quantitation of volume distribution of ZF+ protein in the brain from injected cells from panels **(A,B)**. The gray bar indicates ZF distribution. The black bar indicates the volume of ZF-MSC bolus injection. **(D)** Quantitation of co-localization of ZF protein with NeuN+ cells. ZF/NeuN co-localization decreased from the site of injection in the cortex. **(E)** Quantitation of co-localization of ZF protein with NeuN+ cells. ZF/NeuN co-localization decreased from the site of injection in the striatum. Dashed lines indicate appearance of morphologically distinct neuronal landmarks. Error bars indicate ± standard error of the mean. **(F)** Quantitation of co-localization of ZF protein with NeuN+ cells. ZF/NeuN co-localization decreased in the spinal cord adjacent to the location of the CM administration. DV, dorsal ventral; NeuN+, neuronal nuclei; HA, hemagglutinin. Scale bar = 1,000 μm **(A,B)**, 100 μm **(A1,A2,B1)**.

**FIGURE 4:**
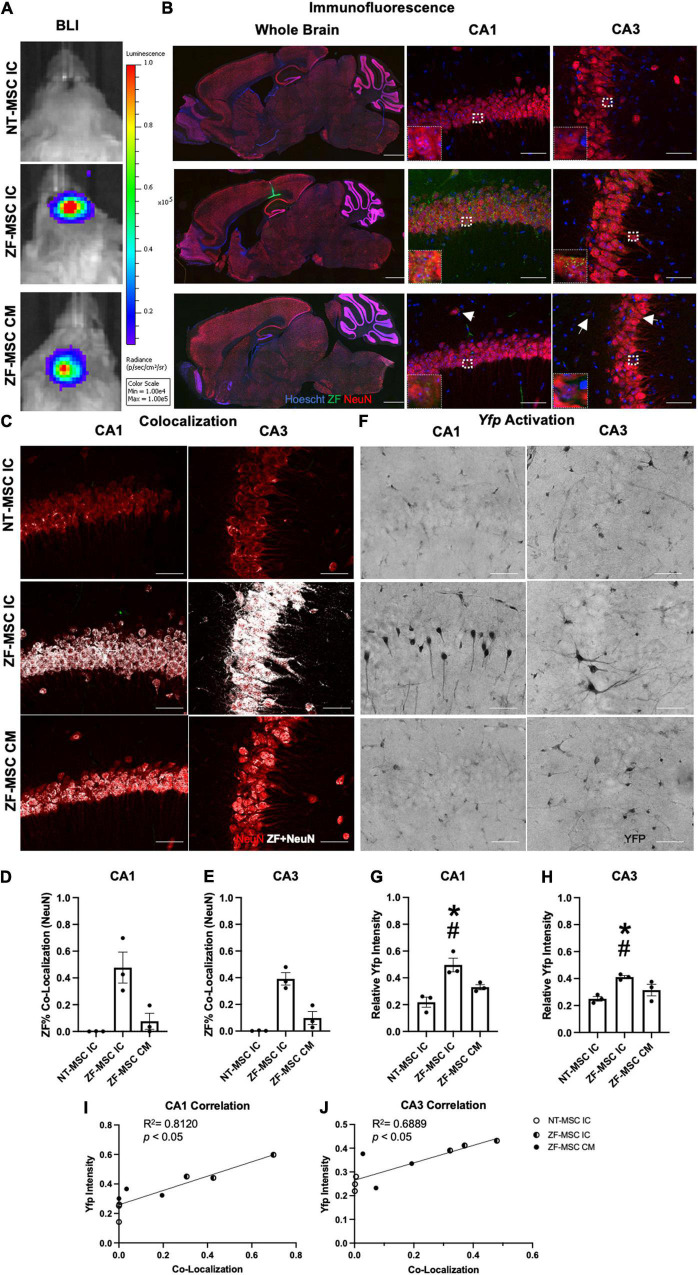
ZF-MSC persist 1 week following injection and demonstrate *In vivo* protein uptake. **(A)** Transduced ZF-MSC are detectable at the surgical site within the brain (ZF-MSC IC) or at the base of the skull (ZF-MSC CM) *via* bioluminescent imaging (BLI) 7 days following administration. **(B)** (Left Column) Bolus of ZF-MSCs (green) were observed within the corpus callosum, dorsal to the hippocampus of IC-injected mice 7 days following transplantation. Scale bar = 1,000 μm. Observable ZF+ (green) puncta are observed within the mature NeuN+ (red) neurons in CA1 (middle column) and CA3 (right column) of the hippocampus in both IC and CM-injected mice. White arrows indicate zinc finger (ZF). Scale bar = 50 μm. Inlet images are 20 μm × 20 μm of indicated region in parent image and depicts ZF+ puncta. **(C–E)** Co-localization of ZF+ (white) were observed in NeuN+ neurons in CA1 **(D)** and CA3 **(E)**. Dots represent individual *Ube3a^+/yfp^* mice. *n* = 3 mice per group. **(C)** Representative images of CA1 (middle row) and CA3 (bottom row) depicting co-localization of ZF+ protein mature neurons. White dots indicate co-localized mCherry+ pixels with red NeuN pixels from images generated in Figure 2B. **(D)** ZF-MSC IC showed a 46.8% co-localization and ZF-MSC CM showed a 7.6% ZF co-localization with NeuN+ neurons across the entire CA1 region of the hippocampus. One-Way ANOVA [LSD *F*_(2, 6)_ = 11.52] followed by a Fisher’s LSD, *p < 0.05 from NT-MSC. **(E)** ZF-MSC IC showed a 39.1% co-localization and ZF-MSC CM showed a 9.8% ZF co-localization with NeuN+ neurons across the entire CA3 region of the hippocampus. One-Way ANOVA [*F*_(2, 6)_ = 26.82] followed by a Fisher’s LSD, *p < 0.05 from NT-MSC. **(F–J)** An increase in UBE3A-YFP was observed in CA1 **(G)** and CA3 **(H)** following IC administration of ZF-MSC. Graphical values normalized to a maternal *Ube3a^Yfp^* positive control. Error bars indicate ± standard error of the mean. Dots represent individual *Ube3a^+/yfp^* mice. *n* = 3 mice per group. **(F)** Representative DAB-labeled images depicting morphologically distinct UBE3A-YFP+ neurons within CA1 (middle row) and CA3 (bottom row) of IC-administered ZF-MSC. Scale bar = 50 μm. **(G)** Quantitation of UBE3A-YFP mean fluorescence intensity of ZF-MSC IC and ZF-MSC CM administration in CA1 of *Ube3a^+/yfp^* mice 1 week following cell injection. ZF-MSC IC significantly increased UBE3A-YFP by 27.8% when compared to NT-MSC IC. One-Way ANOVA [*F*_(2, 7)_ = 14.66] followed by Fisher’s LSD, *p < 0.05 from NT-MSC IC. ^#^*p* < 0.05 from ZF-MSC CM. **(H)** Quantitation of UBE3A-YFP mean fluorescence intensity of ZF-MSC IC and ZF-MSC CM administration in CA3 of *Ube3a^+/yfp^* mice 1 week following cell injection. ZF-MSC IC significantly increased UBE3A-YFP by 16.1% when compared to NT-MSC IC. One-Way ANOVA [*F*_(2, 7)_ = 5.747] followed by Fisher’s LSD, *p < 0.05 from NT-MSC IC. ^#^*p* < 0.05 from ZF-MSC CM. **(I)** YFP expression is strongly and positively correlated with increased ZF co-localization in CA1 from **(D,G)**. [*F*_(1,__7)_ = 11.50] *p* = 0.009, *R*^2^ = 0.8120. **(J)** YFP expression is strongly and positively correlated with increased ZF co-localization in CA3 from **(E,H)**. [*F*_(1,__7)_ = 20.19] *p* = 0.0056, *R*^2^ = 0.6889.

**FIGURE 5:**
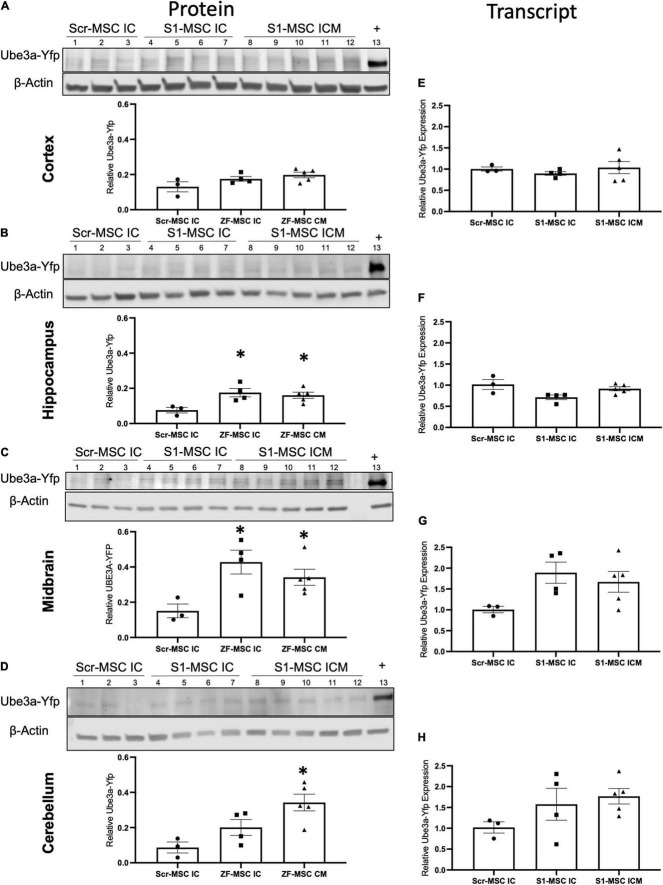
ZF-MSC demonstrate route of administration-dependent activation of *Ube3a^Yfp^* 3 weeks following injection. **(A–D)** Representative Western blots of neuronal subregions with corresponding quantitative graphs. Graphical values normalized to a maternal *Ube3a^Yfp^* positive control. Individual dots represent a single mouse. *n* = 3–5 mice per group. Lanes 1–3 = Scr-MSC IC. Lanes 4–7 = ZF-MSC IC. Lanes 8–12 = ZF-MSC ICM. Lane 13 = Positive YFP control. **(A)** No significant changes in cortical UBE3A-YFP protein expression were found following either ZF-MSC IC or ZF-MSC CM treatment. One-Way ANOVA [*F*_(2, 9)_ = 3.103] followed by Fisher’s LSD, *p < 0.05 to NT-MSC. **(B)** A significant increase in hippocampal UBE3A-YFP protein expression was observed in both ZF-MSC IC and ZF-MSC CM treated mice. One-Way ANOVA [*F*_(2, 9)_ = 6.041] followed by Fisher’s LSD, *p < 0.05 to NT-MSC. **(C)** A significant increase in midbrain UBE3A-YFP protein expression was observed in both ZF-MSC IC and ZF-MSC CM treated mice. One-Way ANOVA [*F*_(2, 9)_ = 7.658] followed by Fisher’s LSD, *p < 0.05 to NT-MSC. **(D)** A significant increase in cerebellar UBE3A-YFP protein expression was observed with ZF-MSC CM treated mice. One-Way ANOVA [*F*_(2, 9)_ = 5.717] followed by Fisher’s LSD, *p < 0.05 to NT-MSC. **(E–G)** Quantitative PCR of neuronal subregions. **(E)** No significant changes in cortical *Ube3a^Yfp^* transcript expression were found following either ZF-MSC IC or ZF-MSC CM administration. One-Way ANOVA [*F*_(3, 10)_ = 0.4581] followed by Fisher’s LSD, *p < 0.05 to NT-MSC. **(F)** No significant changes in hippocampal *Ube3a^Yfp^* transcript expression were found following either ZF-MSC IC or ZF-MSC CM treatment. One-Way ANOVA [*F*_(3, 10)_ = 2.409] followed by Fisher’s LSD, *p < 0.05 to NT-MSC. **(G)** No significant changes were found in midbrain *Ube3a^Yfp^* transcript expression following either ZF-MSC IC or ZF-MSC CM treatment. One-Way ANOVA [*F*_(3, 10)_ = 2.035] followed by Fisher’s LSD, *p < 0.05 to NT-MSC. **(H)** No significant changes were found in cerebellar *Ube3a^Yfp^* transcript expression following either ZF-MSC IC or ZF-MSC CM treatment. One-Way ANOVA [*F*_(3, 10)_ = 1.560] followed by Fisher’s LSD, *p < 0.05 to NT-MSC. Error bars indicate ± standard error of the mean.

**FIGURE 6:**
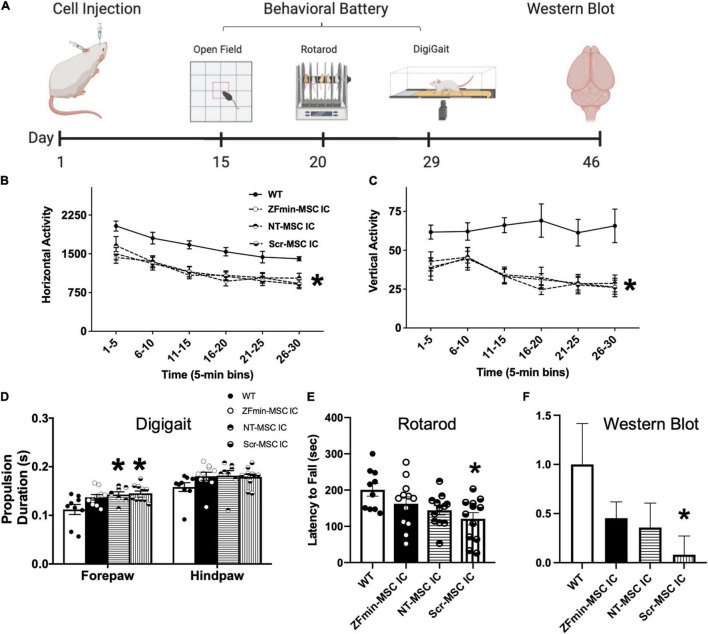
ZF-MSC improves subtle metrics of motor behavior but not gross motor ambulation. **(A)** Schematic of the timeline of cell injections, recovery time, tailored motor behavioral battery for large and small effects in the motor domain in Angelman Syndrome (AS) mice, and tissue collection. **(B)** *Ube3a^mat–/pat+^* mice exhibited typical, robust deficits in horizontal activity and **(C)** vertical activity compared to WT mice. No significant differences were found in locomotion in a novel open field assessing the horizontal and vertical activity using beam breaks in a novel arena. All AS groups, NT-MSC and Scr-MSC and ZF-MSC, regardless of treatment, were different from WT in horizontal activity and vertical activity. Analyses include both males and females. Mean ± standard error of the mean (SEM) is shown. *p < 0.05, two-way repeated-measures ANOVA **(B)** [*F*_(4, 53)_ = 6.286], *p* < 0.001; **(C)** [*F*_(4, 53)_ = 8.475]; *p* < 0.0001, the main effect of the treatment group followed by Sidak’s multiple comparison test. **(D)** Abnormal gait has been shown in *Ube3a^mat–/pat+^* mice and rats. While treadmill walking, the Scr-MSC and NT-MSC treated *Ube3a^mat–/pat+^* mice demonstrated significantly slower forepaw propulsion as compared to WT-littermates. Two-Way ANOVA [*F*_(3, 34)_ = 3.949], *p* < 0.02; followed by Sidak’s Multiple Comparison Test. **(E)** Scr-MSC IC treated *Ube3a^mat–/pat+^* mice had significantly lower latencies to fall off the rotarod compared to WT, corroborating the finding in 6B. Two-Way ANOVA [*F*_(3, 42)_ = 3.798], *p* < 0.02, a Sidak’s Multiple Comparison test could not confirm that performance of ZFmin-MSC IC animals was different from WT or control groups (*p* > 0.05). **(F)** Western Blot analysis of *Ube3a^mat–/pat+^* AS mice showed a significant difference in UBE3A protein expression in NT-MSC and Scr-MSC negative controls compared to WT mice, but not in ZFmin-MSC treated mice. One-Way ANOVA [*F*_(3, 41)_ = 2.092] followed by Fisher’s LSD, *p < 0.05 from WT. Error bars indicate ± standard error of the mean. Dots indicate individual mice. *n* = 9–12 animals per group.

#### Zinc Finger-Mesenchymal Stem/Stromal Cell Intracranial Injections

Prior to surgery, rodent heads were shaved. Mice were anesthetized with isoflurane (2% in O_2_) and placed into a stereotaxic frame (Stoelting, Wood Dale, IL, United States) with ear bars for positioning. The head was aseptically cleaned with betadine and wiped clean with alcohol. A small incision was made in the scalp to allow visualization of the skull, with 1 mm burr holes at −1.5 mm AP and ± 1.25 mm ML relative to bregma. A total of 5 × 10^5^ ZF-MSC or NT-MSC were injected anterior to the hippocampus at a depth of −1.75 using a Hamilton syringe at a rate of 0.5 μl/min with a total volume of 2 μl per side. After waiting an additional 5 min after injection, the burr hole was filled with bone wax and the incision was sutured with 6-0 silk.

#### Zinc Finger-Mesenchymal Stem/Stromal Cell Cisterna Magna Injections

Mice were prepared in a similar manner as noted above. Ear bars were raised until the skull of the mouse was angled at ∼35–40 degrees. A small incision was made at the base of the skull to allow visualization of the CM. The surrounding muscle anterior to the CM was carefully removed to visualize the base of the skull. The Hamilton syringe was placed at the same angle as the skull and was slowly lowered into the CM space with 1 × 10^6^ cells infused at a rate of 0.5 μl/min with a total volume of 4 μl. After waiting an additional 1 min after injection, the incision was sutured closed with 6-0 silk.

#### *In vivo* Imaging Study

Mouse MSCs were transduced by a lentiviral vector carrying the luciferase gene (pCCLc-MNDU3-Luc-PGK-EGFP-WPRE), which allows cells to be visualized in the brains of living mice over time as previously described (Pollock et al., 2016). Bioluminescence was detected in anesthetized animals *via* IVIS Spectrum (Perkin Elmer, Waltham, MA, United States) (Perkin Elmer) 20 min post-intraperitoneal luciferin injection (3 mg/mouse, XenoLight D-Luciferin K+ Salt, Perkin Elmer, Catalog# 122799). Mice were imaged on post-op day 7 prior to the endpoint of the study.

### Molecular Analysis

#### Tissue Extraction

On the last day of study, following cervical dislocation and rapid decapitation brains were rapidly extracted. The cerebral cortex, hippocampus, cerebellum, and midbrain were placed into 1.5 ml tubes and quickly frozen in liquid nitrogen. Tissue was homogenized using a tissue grinder (Kimble Chase, Vineland, NJ, United States, Kontes Pellet Pestle Motor, 749540-0000) and separated for genomic DNA, RNA, or protein.

#### Quantitative PCR Analysis

Tissue was immersed in 350 μl of TRIzol (Zymo Research, Catalog #R2050-1-200) and sonicated using a Dismembrator Sonicator (Thermo Fisher Scientific, Waltham, MA, United States) at setting 3 for 10 s. Sonicated tissue was then centrifuged at 10,000 × *g* relative centrifugal force (rcf) and supernatant was removed and incubated with an equivalent volume of 100% molecular grade ethanol, then RNA was extracted following Direct-Zol RNA Extraction (Zymo Research, San Diego, CA, United States, Catalog #R2053). cDNA was generated using RevertAid Random Hexamer (Thermo Fisher Scientific, Waltham, MA, United States, Catalog # K1691). Ten nanograms of cDNA was probed with TaqMan Universal PCR Master Mix (Thermo Fisher Scientific, Waltham, MA, United States, Catalog #4304437) including probes for Ube3a-Yfp (Thermo Fisher Scientific, Waltham, MA, United States, Catalog #10344-1-AP) and mGAPDH (Thermo Fisher Scientific, Waltham, MA, United States, Catalog MA1-16757) on a StepOne Plus (Applied Biosystems, Foster City, CA, United States). Ube3a-Ats qPCRs were assessed with probes for the 5′ Ube3a-Ats (Ube3a-Ats 5′ F – ACAGAACA ATAGGTCACCAGGTT and Ube3a-Ats 5′ R – AAGCAAGAC TGTTCACCTCAT) and 3′ Ube3a-Ats (Ube3a-ATS 3′ F – CCAA TGACTCATGATTGTCCTG and Ube3a-Ats 3′ R – GTGAT GGCCTTCAACAATCTC). Data are presented as 2^–ΔΔCt^ normalized to Scr-MSC control group.

#### Western Blots

Protein was extracted *via* a standard protein isolation protocol using Pierce RIPA Buffer (Thermo Fisher Scientific, Waltham, MA, United States, Catalog #89901) with Halt Protease and Phosphatase Inhibitor Cocktail (Thermo Fisher Scientific, Waltham, MA, United States, Catalog #78442) and then quantitated using a Bicinchoninic acid assay protein kit (EMD Millipore, Burlington, MA, United States, Kit 71285-3). Forty micrograms of protein lysate was loaded into wells of 4–20% Stacking TGX Gels (BioRad, Hercules, CA, United States, Catalog #4561096EDU), were electrophoresed for 3 h at 100 V, then transferred onto prepared Invitrolon PVDF membrane (Thermo Fisher Scientific, Waltham, MA, United States, Catalog LC2005) and left overnight at 30 V. Membranes were blocked with 10% SEABLOCK salmon sperm (Thermo Fisher Scientific, Waltham, MA, United States, Catalog #37527). The membrane was then transferred to a solution containing rb-GFP (1:1,000, Novus Biologics, Centennial, CO, United States, Catalog #NB600 308) and rb-β-Tubulin (1:2,000, Thermo Fisher Scientific, Waltham, MA, United States, Catalog PA5-26039) with 5% SEA BLOCK in 1% TBST at room temperature for 2 h. Membranes were washed with TBST three times before incubation with 1:2,000 donkey rb-IgG (H + L) 800CW (LI-COR, Lincoln, NE, United States, Catalog #926-32213) and donkey ms-IgG (H + L) 680IR (LI-COR, Lincoln, NE, United States, Catalog #926-68070) in 5% SEABLOCK in 1% TBST for 1.5 h at room temperature. Gels were washed twice in 1% TBST and stored in TBS at 4°C. Bands were imaged using an Odyssey CLx (LI-COR, Lincoln, NE, United States). Optical density of western blots was quantified using ImageJ (NIH, Bethesda, MD, United States). Data are presented as relative percentages against a *Ube3a^matYfp/pat+^* control.

### Imaging

#### Tissue Sectioning

Mice used for immunohistochemistry (IHC) were euthanized using CO_2_ and transcardially perfused with 0.1 M PBS and fresh, ice-cold 4% paraformaldehyde [Avantor (J. T. Baker), Radnor, PA, United States, Catalog S898-07] at pH7.4. All brains were extracted and post-fixed in 4% paraformaldehyde for 36–48 h at 4°C and transferred to 30% sucrose in 0.1 M PBS for 48 h at 4°C. The brains were then cryopreserved in isopropanol on dry ice for 5 min and subsequently stored at ≤−80°C. Serially sagittal sections at 30 μM were obtained using a cryostat (Thermo Fisher Scientific, Waltham, MA, United States), starting from the midline and continuing laterally to a depth of ∼2,700 μm (15 sections in 6 wells), and were stored prior to labeling in a 0.02% sodium azide and 0.1 M PBS solution at 4°C.

#### Co-localization Imaging

Sagittal sections were incubated in a 10% SEABLOCK + 0.1% TBST blocking solution for 1 h prior to incubation with 1:500 rb-mCherry (abcam, Boston, MA, United States, Catalog #Ab167543) and 1:1,000 gp-NeuN (Sigma-Aldrich, St. Louis, MO, United States, Catalog #AB90) at 4°C overnight on gentle agitation. Tissue was then washed with TBST three times for 5 min before incubation with 1:1,000 goat rb-594 and 1:1,000 goat gp-647 AlexaFluor secondary antibodies (Thermo Fisher Scientific, Waltham, MA, United States, Catalog #A111012 and A21450), and then labeled with 1:1,000 Hoechst (Cell Signal Technology, Beverly, MA, United States, Catalog #4082) for nuclear visualization for 5 min. Tissue was then washed with TBST 2× prior to a final TBS 1× wash before mounting and coverslipped with Fluoromount (Sigma-Aldrich, St. Louis, MO, United States, Catalog #F4680-25ML).

#### 3,3′-Diaminobenzidine Labeling

Sagittal sections were labeled with ImmPACT DAB Peroxidase Substrate (Vector Labs, Burlingame, CA, United States, Catalog #SK-4150), utilizing the Vectastain ABC Kit (Vector Labs, Burlingame, CA, United States, Catalog #HP1-26), following the manufactures protocol. Tissues underwent endogenous peroxidase quenching using a 0.3% hydrogen peroxide solution in water for 30 min, followed by immersion in a 10% blocking solution in 0.1% PBST + SEABLOCK Blocking Buffer (Thermo Fisher Scientific, Waltham, MA, United States, Catalog #37527), for 1 h, followed by incubation in primary antibody 1:1,000 rb-GFP (Novus Biologicals, Centennial, CO, United States, Catalog #300-608) and incubated overnight at 4°C. On the second day, tissues were immersed in a biotinylated Goat ms-IgG secondary antibody (Vector Labs, Burlingame, CA, United States, Catalog #BA-9200) solution at a concentration of 1:200 with incubation for 1 h, followed by Vectastain ABC reagent (Vector Labs, Catalog # HP1-26) incubation for 30 min, and concluded with immersion into ImmPACT DAB Peroxidase Substrate (Catalog #SK-4150) at the manufacturers recommended concentration for 8 min. In between each step all tissues were washed using PBST (0.1% Triton) for ∼15 min. Serial sections were mounted onto uncharged slides and coverslipped using Permount Mounting Medium (Thermo Fisher Scientific, Waltham, MA, United States, Catalog #SP15-100).

#### Image Acquisition and Analysis

Images of fluorescent and DAB labels were captured using a Zeiss Observer Z1. Structural specific imaging was performed at 20× for the prefrontal cortex, cerebral cortex, striatum, hippocampus, and cerebellum. Co-localization imaging was completed using the apotome with a 40× objective. Whole-brain imaging was performed at 5× and stitched together using ZEN 2.6. All images were analyzed using ImageJ (NIH, Bethesda, MD, United States). MSC and ZF volume of distribution was calculated using Cavalieri’s volume estimation. The average intensity of YFP was measured in the hippocampus. Images for co-localization were taken with a 40×/0.8 aperture with an inserted ApoSlider at CA1 or CA3. Yfp/NeuN channels were exported and analyzed using a Mander’s Co-Localization Analysis followed by a Costes’ Thresholding through the JaCoP Image J plugin (Bolte and Cordelières, 2006).

### Mouse Behavior

Mice (*n* = 46) were housed in Techniplast cages (Techniplast, West Chester, PA, United States). Cages were housed in ventilated racks in a temperature (68–72°F) and humidity (∼25%) controlled colony room on a 12:12 light/dark cycle. Standard rodent chow and tap water were available *ad libitum*. In addition to standard bedding, a Nestlet square, shredded brown paper, and a cardboard tube (Jonesville Corporation, Jonesville, MI, United States) were provided in each cage for enrichment. The order and age of testing were as follows: (1) Open field at 8 weeks of age, (2) Rotarod at 9 weeks of age, and (3) *DigiGait* at 10 weeks of age.

#### Open Field

General exploratory locomotion in a novel open field arena was evaluated as previously described (Ku et al., 2016; Berg et al., 2018). Briefly, each subject was tested in a VersaMax Animal Activity Monitoring System (Accuscan, Columbus, OH, United States) for 30-min in a ∼30 lux testing room.

#### Rotarod

Motor coordination, balance, and motor learning were tested with an accelerating rotarod (Ugo Basile, Gemonio, Italy) as previously described (Williams, 2010). Mice were placed on a rotating cylinder that slowly accelerated from 5 to 40 revolutions per min over 5 min. Mice were given three trials per day with a 60-min inter-trial rest interval and tested for 3 consecutive days for a total of nine trials. Performance was scored as latency to fall off the cylinder with a maximum latency of 5 min.

#### Gait Analysis

Treadmill gait analysis was performed using the DigiGait™ system (Mouse Specifics Inc., United States). Mouse paws were painted with non-toxic red food coloring to augment dark green paw tattoos that generated conflict in DigiGait™ analysis one minute prior to introduction to the walking chamber to reliably capture the entire paw surface area. For each mouse, videos of ∼5 sec duration of all sessions were analyzed using the DigiGait™ Imaging and Analysis software v12.2 (Mouse Specifics Inc., United States). Contrast filters were determined on a mouse-by-mouse case to facilitate consistent recognition of all four paws. All analysis was conducted in a single session by experimenter blind to genotype. Data was averaged between left and right paws for fore and hind paws.

### Rhesus Monkeys

**FIGURE 7:**
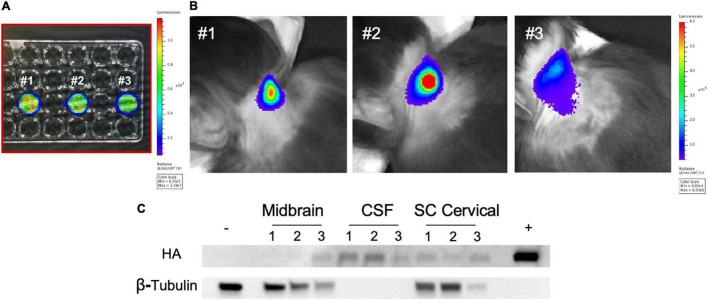
Intrathecal administration of autologous rhMSC in young rhesus monkeys. **(A)** Autologous rhMSC were collected per standard protocol and cells transduced with a lentiviral vector expressing firefly luciferase. rhMSC were confirmed to express luciferase by bioluminescence imaging (BLI) prior to intrathecal injection. Numbers reflect transduced rhMSC from monkey #1, monkey #2, and monkey #3. **(B)** BLI was performed immediately following intrathecal administration of rhMSC expressing firefly luciferase according to established protocols. The location of the firefly luciferase-expressing autologous rhMSC post-injection are shown in representative images from each rhesus monkey, consistent with findings in other cell and gene transfer studies where BLI has been used (Tarantal et al., 2009; Tarantal and Lee, 2010). **(C)** Western blot shows the presence of zinc finger (ZF) 3 weeks following injection. ZF protein was delectable in the CSF (three animals), midbrain (one animal), and spinal cord (two animals). Historical whole brain lysate from non-treated rhesus monkeys was used as a negative control. ZF-MSC lysate used as a positive control. Numbers reflect animal number as described in **(A)**.

All animal procedures conformed to the requirements of the Animal Welfare Act and protocols were approved prior to implementation by the Institutional Animal Care and Use Committee at the University of California, Davis. Three young rhesus monkeys (*Macaca mulatta*) (∼3 months of age; 1 kg) were included in these studies. Autologous rhesus monkey bone marrow-derived MSCs (rhMSC) were obtained and cells were grown in culture and transduced according to established published protocols (Figure 7A; Lee et al., 2004, 2006). Luciferase expression was confirmed with bioluminescence imaging (BLI) prior to *in vivo* administration (IVIS ^®^ Spectrum *in vivo* Imaging System with Living Image Software; PerkinElmer). A total of 1 × 10^6^ rhMSC expressing firefly luciferase were administered to each of the telazol-sedated animals (5–8 mg/kg) using an established IT approach (0.5 ml CSF removed using a 25 g needle and 3 ml syringe, then the syringe was changed to inject ∼0.5 ml rhMSC slowly) under aseptic conditions (Hordeaux et al., 2019). BLI was immediately performed post-injection of rhMSC and after the intravenous administration of D-Luciferin (100 mg/ml) according to established methods (Figure 7B; Tarantal et al., 2009; Tarantal and Lee, 2010). Animals were monitored throughout the day then daily physical signs were assessed with peripheral blood sample collection (∼2–3 ml) and body weight weekly [complete blood counts, chemistry panels; all within normal limits (data not shown)]. At 3 weeks post-administration animals were sedated with ketamine, blood samples and CSF collected, then euthanized with an overdose of pentobarbital for tissue harvests according to established methods. Multiple sections of the brain (right and left frontal, parietal, temporal, occipital lobes; hippocampus; right and left cerebellum; midbrain) and spinal cord (cervical, thoracic, and lumbar) were collected and placed in sterile tubes and quick frozen over liquid nitrogen and stored at ≤−80°C until assay. The presence of ZFs was evaluated following the same western blot techniques described above.

#### Statistics

All statistical analysis was performed using GraphPad Prism 7 with an alpha level equal to 0.05. All molecular and histological data was analyzed using an analysis of variance or Student’s *t*-test where appropriate to assess differences between groups. When appropriate, a Fisher’s Least Significant Difference *post hoc* test was also performed.

## Results

### Engineered Mouse Mesenchymal Stem/Stromal Cell Can Function as Biofactories for Zinc Finger and Transcription Activator-Like Effectors

The initial ZF protein construct contained an N-terminal maltose-binding protein for protein purification, the 10-aa transduction domain of the HIV-transactivator protein (TAT, residues 48–57) as a cell-penetrating peptide, a mCherry red fluorescent protein to aid in and visualization, a Hemagglutinin (HA) epitope tag for detection, and a SV40 nuclear localization signal to ensure nuclear delivery following cellular transduction. The TAT cell-penetrating peptide had been previously shown to deliver other proteins to the mouse brain following intraperitoneal injection (Gong et al., 2006; Wu et al., 2015). The C-terminus of the protein consisted of the ZF array and KRAB repressor domain. We replaced the maltose binding protein with an IgK Signal Peptide as a secretion peptide and altered the original TAT peptide with a furin-resistant TAT (TATk) for improved protein stability (Flinterman et al., 2009) and subcloned into a lentivirus compatible vector This specific orientation of IgK-TATk-mCherry-HA-NLS-ZF-KRAB would allow for active secretion of the fully translated protein into the extracellular space of a cell, followed by uptake into neighboring cells through the TAT peptide, and then localization to the nucleus to silence the *Ube3a-Ats* (Figure 1A). We transduced mouse bone marrow derived MSCs with this ZF secretion vector (ZF-MSC) and found secretion of the full length 67-kDa ZF protein in the ZF-MSC-conditioned media (Figure 1B). We incubated ZF-MSC-conditioned media with Neuro2A cells and observed co-localization of mCherry-tagged ZF in recipient cells within 24 h (Figure 1C, right panel). Additionally, engineered MSCs were able to secrete ATF of different sizes such as a smaller 62-kDa ZF and a 120 kDa TALE (Supplementary Figures 1A,B). We evaluated alterations in MSC phenotype following transduction and found that both a ZF-MSC and a scramble ZF MSC (Scr-MSC) demonstrated typical MSC markers as assessed by flow cytometry and did not affect proliferation when compared to non-transduced MSC (NT-MSC) controls (Figures 1D,E and Supplementary Figures 2, 3).

### *In vitro* Functional Validation of Secreted Zinc Finger Protein

Our designed ZF binds to the putative *Ube3a-Ats* promoter and represses expression of this long non-coding antisense RNA, allowing for expression of the intact paternal *Ube3a* gene (Figure 2A). We evaluated the biological functionality of an MSC-mediated secreted ZF in a series of *in vitro* assays. The therapeutic potential of MSCs is thought to be mediated by two mechanisms of action—microtubule conjugation with neighboring cells to allow for direct transfer of cargo or secretion of bioactive molecules that act in a paracrine manner (Liang et al., 2014). The paracrine mechanism of action was evaluated through co-incubation of engineered MSCs with recipient Neuro2A mouse neuroblastoma cells in Transwell Assays (Figure 2B). Both our ZF-MSC and an epitope-free, ZFmin-MSC variant, demonstrated reduction in *Snrpn* expression, the proximal gene to where the ZF binds, following co-incubation with Neuro2A cells. We observed that the Neuro2As treated with the purified protein version of ZF demonstrated a significant 28.6% reduction of *Snrpn* transcript expression 14 h following treatment compared to a NT-MSC conditioned media *via* RT-qPCR (Figure 2C). This effect was ameliorated at 24 h (Figure 2D) and 48 h (Figure 2B) compared to NT-MSC control. In comparison, we did not observe a significant change in *Snrpn* expression following a 24 h incubation with either ZF variant (Figure 2D). At 48 h, a significant 15% reduction in *Snrpn* was observed in ZFmin-MSC treated cells but no significant changes were detected with ZF-MSC (Figure 2E). At 72 h, significant *Snrpn* reduction was observed in ZF-MSC (24.7%) and ZFmin-MSC (20.2%) treated cells relative to the NT-MSC control (Figure 2F).

Next, we evaluated secreted ZF functionality in primary cortical cultures from the *Ube3a^+/yfp^* reporter mouse model. The paternal *Ube3a* is fused to a yellow fluorescent protein (*Yfp*) reporter and is silenced in mature neurons (Dindot et al., 2008). We incubated conditioned media from ZF-MSC, ZFmin-MSC, or purified ZF in primary cortical neurons harvested from E15.5 *Ube3a^+/yfp^* pups for 24 h (Figure 2E). Immunocytochemistry (ICC) of treated primary cortical neurons revealed an increase of YFP expression in all treatment groups compared to Scr-MSC conditioned media and no significant differences in YFP expression between purified ZF treatment and ZF-MSC variants following a 24 h incubation with conditioned media (Figures 2F,G). Western blot analysis demonstrated increased UBE3A-YFP in treated samples (Figure 2H). Together, these data demonstrate the functionality of secreted ZF from MSCs in both immortalized and primary mouse cell lines to activate paternal *Ube3a* expression.

### Robust Protein Uptake and Biological Effect of Secreted Zinc Finger Protein

We evaluated our platform *in vivo* to understand the kinetics and distribution of a cell secreted ZF across different routes of administration. Initially, a total of 1 × 10^6^ ZF-MSC were injected intracranially (IC) into the striatum or *via* the CM in wild-type (WT) FVB mice. We observed injected MSCs at the site of IC injection (Figure 3A) and presence of secreted ZF protein in the extracellular space of IC and CM injections (Figures 3A–C). ZF-MSC IC protein appeared to distribute from the injection site as measured from both the dorsal (cortex) and ventral (striatum) plane. A Mander’s Colocalization Coefficient Analysis followed by a Costes’ automatic thresholding revealed a gradient-specific decline of protein uptake in NeuN+ cells that are more distally anterior and posterior from the injection site (Figures 3D,E). Interestingly, ZF-MSC CM protein also demonstrated a gradient-dependent distribution of ZF protein uptake from the site of CM injection (Figure 3F).

Following the confirmation of *in vivo* secretion of ZF protein, 1 × 10^6^ ZF-MSC were bilaterally injected intracranially dorsal to the region of interest in AS, the hippocampus, or *via* the CM in the *Ube3a^+/yfp^* reporter mouse. BLI demonstrated that transduced MSCs persisted at least 1 week following injection (Figure 4A). For IC delivery, IHC analysis revealed that ZF-MSCs were observed along the needle tract and the white matter in the corpus callosum in IC-injected mice (Figure 4B and Supplementary Figure 4). MSCs were found in the third ventricle and the cerebellum (Supplementary Figure 4). In contrast, MSCs delivered *via* the CM were observed along the surface of the cortex, cerebellum, spinal cord, and hypothalamus of the mouse brain (Supplementary Figure 5). Secreted ZF protein was observed in NeuN+ cells within the CA1 and CA3 regions of the hippocampus following both routes of administration (Figure 4C). A Mander’s Colocalization Coefficient Analysis followed by a Costes’ automatic thresholding revealed that IC delivery resulted in 46.8% of CA1 and 39.1% of CA3 NeuN+ areas co-labeled with ZF protein as compared to 7.6% and 9.8%, respectively, in mice administered MSC *via* CM delivery (Figures 4D,E).

### Zinc Finger-Mesenchymal Stem/Stromal Cell Activates Paternal *Ube3a^Yfp^* Expression in the *Ube3a^+/Yfp^* Mouse

Yellow fluorescent protein activation was observed in morphologically distinct pyramidal neurons in the hippocampus of ZF-MSC administered IC mice using 3,3′-Diaminobenzidine (DAB) IHC 1 week post-delivery (Figure 4F). Relative YFP mean intensities of IC injected ZF-MSC treated mice demonstrated a significant 27.8% increase (Figure 4G) in CA1 and a significant 16.1% increase (Figure 4H) in CA3 of the paternally imprinted UBE3A-YFP protein in ZF-MSC treated *Ube3a^+/yfp^* mice when compared to the NT-MSC controls. No effect was observed in the CM group at the 1-week time point (Figures 4G,H). We observed a positive correlation between ZF co-localization and YFP intensity in CA1 (Figure 4I), and CA3 (Figure 4J).

Paternal *Ube3a^Yfp^* activation was further evaluated in discrete brain regions through molecular assays. We observed a route of administration-specific divergence in which neuronal subregions showed alterations in *Ube3a^Yfp^* expression. *Ube3a^+/yfp^* mice administered ZF-MSC IC or ZF-MSC CM did not demonstrate a difference in UBE3A-YFP protein expression in the cortex compared to Scr-MSC IC groups (Figure 5A). In the hippocampus, there was a significant 9.9% and 8.5% increase in UBE3A-YFP protein of ZF-MSC IC and ZF-MSC CM, respectively (Figure 5B). In the midbrain, there was a significant 27.6% and 19% increase in the UBE3A-YFP protein of ZF-MSC IC and ZF-MSC CM treated mice, respectively (Figure 5C). In the cerebellum, there was a significant 25.5% increase in UBE3A-YFP protein of ZF-MSC CM treated mice, but no observable effect in ZF-MSC IC mice (Figure 5D). No significant effects were observed in *Yfp* transcript expression (Figures 5E–H). This transcriptional data was further corroborated by no observable changes in either the 3′ or 5′ Ube3a-Ats transcript (Supplementary Figure 6). Together these data demonstrate *in vivo* activation of *Ube3a^Yfp^* protein, but not transcript 3 weeks after injection.

### Zinc Finger-Mesenchymal Stem/Stromal Cell Attenuates Motor Deficits in the *Ube3a^mat–/pat+^* Angelman Syndrome Mouse

Following activation of paternal *Ube3a^Yfp^* in the *Ube3a^+/yfp^* mice, we utilized IC delivery of ZFmin-MSC for behavioral assessments in the *Ube3a^mat–/pat+^* AS mouse model (Jiang et al., 1998). Motor deficits have been consistently reported across mouse models of AS, such as dysfunction on the rotarod and reduced activity in a novel arena (Huang et al., 2013; Born et al., 2017; Sonzogni et al., 2018), thus we focused on a motor phenotyping battery for functional rescue. Six-week-old *Ube3a^mat–/pat+^* mice underwent bilateral IC injection of 1 × 10^6^ ZFmin-MSC and were allowed to recover for 2 weeks following surgery before undergoing a motor-tailored behavioral battery (Figure 6A). All AS groups regardless of treatment differed from WT in horizontal activity (Figure 6B) and vertical activity (Figure 6C), a hallmark of AS pathology (Adhikari et al., 2021). In gait analysis, NT-MSC and scrambled ZF MSC) treated *Ube3a^mat–/pat+^* mice showed deficits in forelimb propulsion as time in the propulsion phase was significantly increased compared to WT littermates (Figure 6D) whereas ZFmin-MSC treated mice were indistinguishable from WT mice. Scr-MSC treated mice also exhibited behavior that is typical of *Ube3a^mat–/pat+^* mice and rats with reduced latencies to fall on an accelerating rotarod assay compared to WT-littermates (Huang et al., 2013; Born et al., 2017; Berg et al., 2020). Both NT-MSC and ZFmin-MSC treated mice demonstrated an intermediate effect; while there was an overall genotypic effect (Figure 6E), performance was not different from WT or Scr-MSC groups. We observed a significant reduction in UBE3A protein in Scr-MSC treated mice as compared to WT littermates; however, ZFmin-MSC and NT-MSC were not significantly different from either of those groups (Figure 6F).

### Young Rhesus Monkeys Demonstrate Presence of Secreted Zinc Finger

Autologous rhMSC were co-transduced to express firefly luciferase and secrete ZF (ZF-rhMSC) (Figures 7A,B). ZF-rhMSC (1 × 10^6^) were injected under aseptic conditions using an established IT approach and were well-tolerated for the duration of the 3-week study. CBCs and chemistry panels within normal limits (**data not shown**). Tissues were collected 21 days post-administration and assessed for the presence of ZF protein. Secreted ZF protein was detected in the CSF (2 of 3 animals), midbrain (1 of 3 animals), and spinal cord (3 of 3 animals) (Figure 7C).

## Discussion

Mesenchymal stem/stromal cells have been used as a delivery system for bioactive molecules in cross correction approaches (Meyerrose et al., 2010; Damasceno et al., 2020) and gene modifying molecules such as shRNAs (Olson et al., 2012). Here, we adapted the innate paracrine abilities of MSCs to secrete artificial transcription factors of various sizes that remain bioactive *in vivo*. We report the use of MSCs to secrete a bioactive ZF to alter *Ube3a* expression in AS mouse models and demonstrate detectable distribution in midbrain and spinal cord of rhesus monkeys. We sought to evaluate if the secretable ZF protein would retain its ability to distribute widely in the brain using multiple routes of administration (surgical IC, CM, or IT).

We report co-localization of secreted ZF protein with mature neurons in the mouse hippocampus 1 week following either an IC injection that was proximal to the hippocampus or a more distal CM injection. Direct ZF-MSC injection into the parenchyma of the mouse brain demonstrated a passive diffusion of secreted protein from the bolus of cells that was likely taken up by neighboring cells. Interestingly, secreted ZF was detectable in the deeper, midbrain in the mouse and monkey. ZF-MSCs were detectable within the spinal cord of ZF-MSC treated mice upon CM delivery and thus likely a source of ZF protein in the CSF. This was also shown by detection of the ZF protein within the spinal cord and CSF of rhesus monkeys. It is not well understood how a TAT-fused peptide is able to penetrate deeper, subcortical regions that are distal from the arachnoid space as the flow of CSF is canonically thought to originate in the choroid plexus and then flow into the lateral ventricles, the third ventricle, the cisterns, the arachnoid space, and then empty into the arachnoid villi (Brinker et al., 2014). Interestingly, Papisov et al. (2012) demonstrated that lumbar IT injection of large macromolecules rapidly distribute into the CSF and are taken up into the tissue, potentially aided by pulsatile movements of arteries in the leptomeningeal space. Mazur et al. (2019) demonstrated that IT administration of macromolecules such as antisense oligonucleotides penetrate subcortical regions such as the hippocampus and thalamus of the brain more slowly compared to cortical spaces and hypothesized that this may be mediated by exchange of proteins into the perivascular spaces that have deeper brain access (Barua et al., 2014). We and others have previously shown that the inclusion of a TAT-peptide allows for robust penetration of recombinant proteins along the brain parenchyma (Schwarze et al., 1999; Kilic et al., 2002, 2003; Doeppner et al., 2010; Ye et al., 2011; Wu et al., 2015) and this likely enhances the ability for a secreted protein to have a broad distribution along the neuroaxis. Furthermore, MSCs are well known to have a vast secretome that is mediated through the release of exosomes. The present cell delivery system is designed to secrete naked protein, however, further evaluation of the potential contribution of exosomal delivery of ATFs is of interest for future investigation.

Zinc finger-mesenchymal stem/stromal cell secreted protein and purified ZF protein (Bailus et al., 2016) demonstrated differing pharmacokinetic profiles *in vitro*. Purified ZF induces an immediate reduction of *Snrpn* expression that quickly tapers off due to the relatively short half-life of ZF proteins (Ramakrishna et al., 2013; Bailus et al., 2016). In comparison, ZF-MSC are able to induce a sustained reduction of *Snrpn* within 48 h of incubation. This delayed effect is likely due to the time necessary for sufficient ZF protein to be produced and secreted by MSC. We observed a significant increase in hippocampal UBE3A-YFP in *Ube3a^+/yfp^* mice administered ZF-MSC by the IC route within 1 week of injection. However, no effect was observable in the CM group. We found that the amount of ZF co-localization with hippocampal neurons was strongly correlated with increases in UBE3A-YFP, suggesting that sufficient exposure to ZF protein is necessary to elicit a biological effect—in agreement with our *in vitro* and *in vivo* data that demonstrates a slower ZF accumulation in regions that are more distal to the CM.

We also observed a divergence in the brain regions that are amenable to alterations of gene expression concerning the different administration routes studied. At 3 weeks, mice treated by either delivery route expressed more UBE3A-YFP in the midbrain and hippocampus, whereas only CM delivery of ZF-MSC resulted in the expression of more UBE3A-YFP in the cerebellum. It is interesting to note that ZF-MSCs are highly motile in the brain following either route of administration—a similar phenomenon that has been observed in other tissues (Chen et al., 2015; Argibay et al., 2017). Intracranial-delivered ZF-MSC appeared to travel along the corpus callosum toward CSF-rich spaces such as the lateral ventricles where the cells could be detected throughout areas in the brain such as the third ventricle and the cerebellum. CM delivered ZF-MSC were detectable in the spinal cord, cerebellum, and along the apical or basal regions of the cortex. The ability for ZF-MSCs to migrate and embed in other regions of the mouse brain may promote a localized accumulation of ZF in CSF-rich recesses such as the lateral ventricles to induce paternal *Ube3a^Yfp^* activation in subcortical regions of the mouse brain. The ability for MSCs to travel beyond their site of injection may further potentiate their ability to act as roaming biofactories within the CNS. Data supports that a less invasive, non-surgical CM injection effectively delivers engineered MSC in local and distal regions of the brain. Additionally, this observation is confirmed in studies that tested the feasibility, safety, and tolerability of IT administration of ZF-rhMSCs in young rhesus monkeys. These cells were well-tolerated and did not result in any adverse events for the duration of the study. Importantly, secreted ZF protein was detectable within the CSF, midbrain, and spinal cord in rhesus. Previous examples in the gene therapy field indicate that transitioning directly from mouse studies to human clinical trials is fraught with species and technical limitations, including anatomical and physiological differences between brain structure and size. This concern necessitates the use of a larger primate species such as the rhesus monkey that simulates human anatomy and physiology. Our data support that our system allows for delivery of ZF to the brain in a safe, non-surgical manner that may allow for administration of engineered MSC into the CSF to promote a biological effect.

Movement disorders affect nearly every individual with AS (Tan et al., 2011; Wheeler et al., 2017; Keute et al., 2020). Previous work by Silva-Santos et al. (2015) demonstrated that 70% restoration of *Ube3a* at postnatal day 21 in an inducible *Ube3a* mouse resulted in only a partial recovery of rotarod performance, suggesting that there was a critical window of treatment at 21–42 days following birth. This was further corroborated by recent work by Wolter et al. (2020) showing prenatal intervention in *Ube3a-Ats* expression resulted in attenuation of disease phenotypes and restoration of *Ube3a* expression. In our study, ZF-MSCs were able to induce improvements in forepaw propulsion and latency to fall on the accelerating rotarod in *Ube3a^mat–/pat+^* mice when treated on post-natal day 42. Our results suggest that ZF-MSC treatment improved subtle motor control as mice were rapidly placing their paws onto the ground or improving muscular weakness as less time was required for forelimb strides, which was significantly elevated in *Ube3a^mat–/pat+^* relative to WT littermate controls. It is interesting to note that an intermediate effect was also observed for NT-MSC treated mice in the latency to fall on the accelerating rotarod task. MSCs secrete several favorable trophic factors including brain-derived neurotrophic factor and noggin that can improve phenotypic outcomes in mice (Lu et al., 2003). While our scrambled controls did not induce any improvements in accelerod, we cannot discount the effect that unaltered MSCs alone may have on motor phenotypes. Importantly, we only observed an intermediate effect in *Ube3a* expression in ZFmin-MSC following IC surgeries, suggesting that an epitope-free form of the ZF has a favorable pharmacodynamic profile for up to 29 days.

Previous work by our group and others suggest that the biologic effect of a KRAB-fused ATF is transient transcriptional repression (Gilbert et al., 2014) and is contingent on presence of the gene modifying protein. UBE3A has a half-life of ∼61 h (Olabarria et al., 2019). We did not observe a detectable presence of ZF-MSC 3 weeks following injection through either route of administration in mice, suggesting that injected MSCs were rapidly cleared from the brain parenchyma. Thus, the lack of *Ube3a^Yfp^* transcriptional expression may be indicative of a tapering of the therapeutic effect as the ZF protein clears while elevated UBE3A-YFP protein expression persists for some time post-injection. Our current platform may benefit from continued advances in defining gene modifiers that induce durable epigenetic markers that result in sustained alterations in gene expression (Amabile et al., 2016; O’Geen et al., 2019; Nuñez et al., 2021). Temporal secretion of such gene modifiers that are capable of widespread coverage within the CNS through cellular biofactories may offer an ideal delivery vehicle for “hit-and-run” epigenetics.

## Data Availability Statement

The raw data supporting the conclusions of this article will be made available by the authors, without undue reservation.

## Ethics Statement

Animal studies were approved by the UC Davis Institutional Animal Care and Use Committee.

## Author Contributions

PD, AT, JS, DS, and KF: conceptualization. PD, FF, AT, JS, DS, and KF: methodology. PD, JH, UB, RL, DC, JW, KT, MP, AA, NC, SP, HO’G, SL, AT, MM, CL, and KF: investigation. AT, JN, JS, and KF: resources. PD: writing—original draft. PD, JH, AT, JN, JS, DS, and KF: writing—review and editing. PD and JH: visualization. KF: supervision and project administration. AT, JN, DS, and KF: funding acquisition. All authors contributed to the article and approved the submitted version.

## Conflict of Interest

The authors declare that the research was conducted in the absence of any commercial or financial relationships that could be construed as a potential conflict of interest.

## Publisher’s Note

All claims expressed in this article are solely those of the authors and do not necessarily represent those of their affiliated organizations, or those of the publisher, the editors and the reviewers. Any product that may be evaluated in this article, or claim that may be made by its manufacturer, is not guaranteed or endorsed by the publisher.

## Supplementary Material

Supplementary Figure 1 Transduced MSCs are able to secrete full-length zinc finger and transcription activator-like effector proteins, related to Figure 1. **(A)** Full-length 62-kDa ZF is detectable in conditioned media from ZF-MSCs but absent in isogenic NT-MSCs. Plasmid map indicates size of secretion ZF transgene and predicted protein size. **(B)** Full-length 120-kDa TALE is detectable in conditioned media from TALE-MSCs but absent in isogenic NT-MSCs. Plasmid map indicates size of secretion TALE transgene and predicted protein size.

Supplementary Figure 2 Transduced mouse BM-MSCs demonstrate positive canonical MSC markers CD44b and negative for CD11b, CD34b, and CD106, related to Figure 1. **(A)** Isotype FITC negative control. **(B)** NT MSC, Scr-MSC, and ZF-MSCs are negative for immune marker CD11b. **(C)** NT MSC, Scr-MSC, and ZF-MSCs are negative for hemopoietic stem cell marker CD34b. **(D)** NT MSC, Scr-MSC, and ZF-MSCs are positive for MSC surface marker CD44b. **(E)** NT MSC, Scr-MSC, and ZF-MSCs are negative for vascular adhesion marker CD106.

Supplementary Figure 3 Transduced mouse BM-MSCs demonstrate positive canonical MSC markers CD29b and Sca1 and negative for CD31, CD45, and CD105, related to Figure 1. **(A)** Isotype PE negative control. **(B)** NT MSC, Scr-MSC, and ZF-MSCs are positive for MSC surface marker CD29b. **(C)** NT MSC, Scr-MSC, and ZF-MSCs are negative for platelet endothelial adhesion molecular marker CD31. **(D)** NT MSC, Scr-MSC, and ZF-MSCs are negative for differentiated hemopoietic cell marker CD45b. **(E)** NT MSC, Scr-MSC, and ZF-MSCs are negative for vascular endothelial marker CD105. **(F)** NT MSC, Scr-MSC, and ZF-MSCs are enriched for mouse MSC marker Sca-1.

Supplementary Figure 4 Intracranial injection of ZF-MSC demonstrated motility from the parenchyma, related to Figure 4. **(A)** NT-MSC IC was observable within the mouse brain one week following injection and detected moving towards the lateral ventricles along the corpus callosum **(A1)**. The white box denotes inlet of an image in panel **(A1)**. Dashed white lines indicate the presence of injected MSC due to distinct morphology in contrast to mature NeuN+ (red) neurons and nuclear Hoechst (blue) labeling. **(B)** ZF-MSC IC (green) was observable within the mouse brain following injection and detected moving along the corpus callosum towards the lateral ventricles **(B1)**. A white box denotes an inlet of image in panel **(B1)**. **(C)** ZF-MSCs IC were detectable across multiple regions of the brain, including the forebrain **(C1)**, site of injection along the corpus callosum **(C2)**, anterior to the hippocampus **(C3)**, along the third ventricle–ventral **(C4)** and posterior **(C5)** to the hippocampus, in the visual cortex **(C6)**, along with the cerebellum **(C7,C8)**, and within the lateral ventricles **(C9)**. Scale bar = 500 μm **(A–C)**, 50 μm **(A1,B1,C1–C9)**.

Supplementary Figure 5 Cisterna magna injection of ZF-MSC demonstrated motility to the CSF one week following injection, related to Figure 4. **(A)** Cisterna magna (CM) injected ZF+ (green) were detectable along the somatosensory cortex **(A1)** and were distinct from surrounding NeuN+ (red) neurons and other Hoechst (blue) labeled cells. **(B)** ZF-MSC injected via the CM were detectable across multiple regions including the cortex **(B1)**, cerebellum **(B2–B4)** and along hypothalamic regions **(B5–B6)**. **(C)** ZF-MSC injected via the CM were detectable within the mouse spinal cord. Scale bar = 500 μm **(A–C)**, 50 μm **(A1,B–B6)**.

Supplementary Figure 6 YFP transcript expression is not altered with response to ZF, related to Figure 5. **(A)** No observable changes in the 3′ Ube3a-Ats transcript was observed in the cortex, hippocampus, midbrain, or cerebellum in 3-weeks post ZF-MSC treatment in *Ube3a^+/yfp^* mice. **(B)** No observable changes in the 5′ Ube3a-Ats transcript was observed in the cortex, hippocampus, midbrain, or cerebellum in 3-weeks post ZF-MSC treatment in *Ube3a^+/yfp^* mice. Error bars indicate ± standard error of the mean. Dots indicate individual mice.
