# Supplementary figures and images for: An in vivo Cell-Based Delivery Platform for Zinc Finger Artificial Transcription Factors in Pre-clinical Animal Models
Source: Front Mol Neurosci. 2022 Jan 27;14:789913. doi: 10.3389/fnmol.2021.789913 (PMC8829036; doi:10.3389/fnmol.2021.789913)

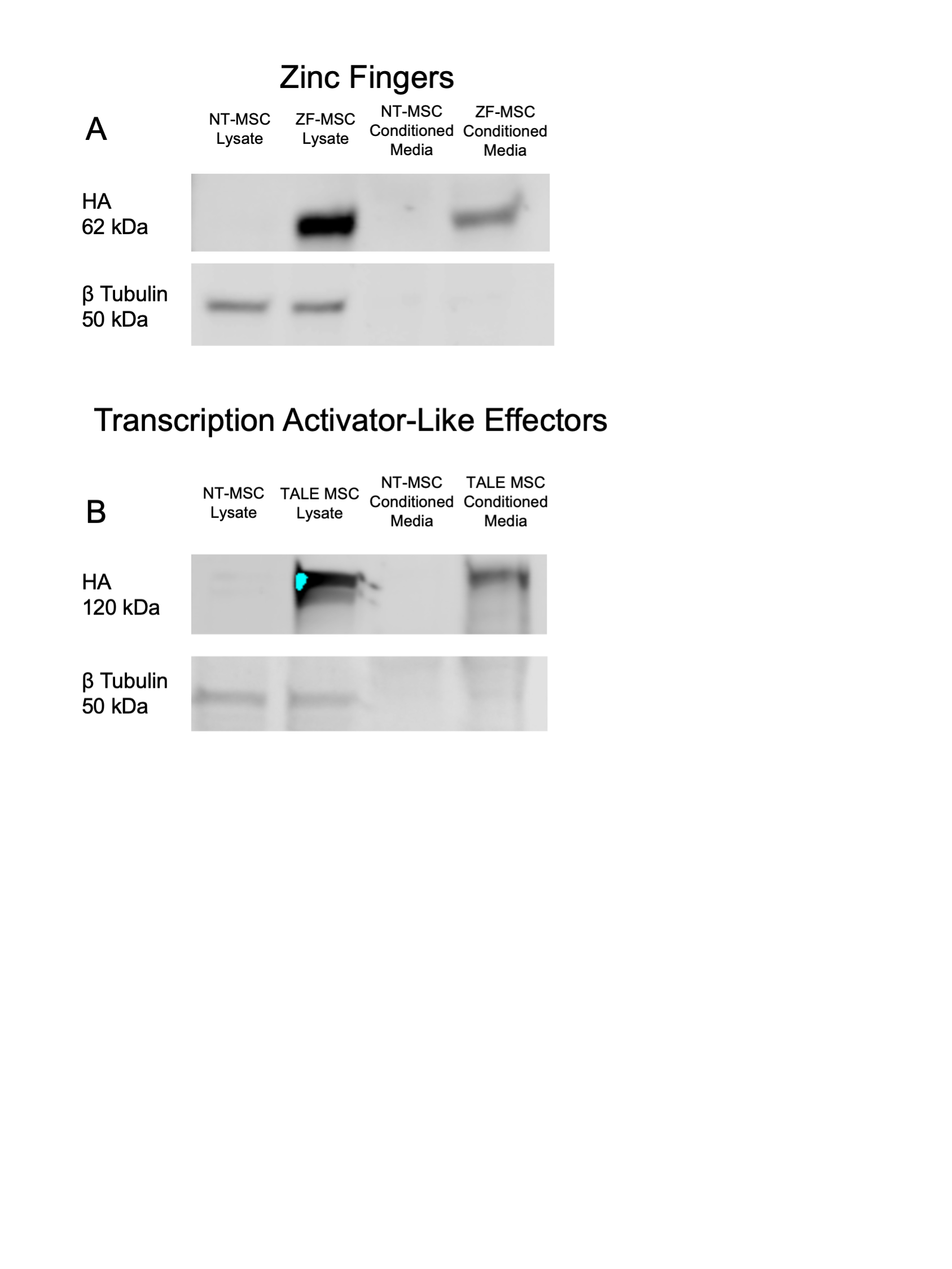

Supplement: Supplementary Figure 1 — Transduced MSCs are able to secrete full-length zinc finger and transcription activator-like effector proteins, related to Figure 1. (A) Full-length 62-kDa ZF is detectable in conditioned media from ZF-MSCs but absent in isogenic NT-MSCs. Plasmid map indicates size of secretion ZF transgene and predicted protein size. (B) Full-length 120-kDa TALE is detectable in conditioned media from TALE-MSCs but absent in isogenic NT-MSCs. Plasmid map indicates size of secretion TALE transgene and predicted protein size. [file Image_1.TIFF]

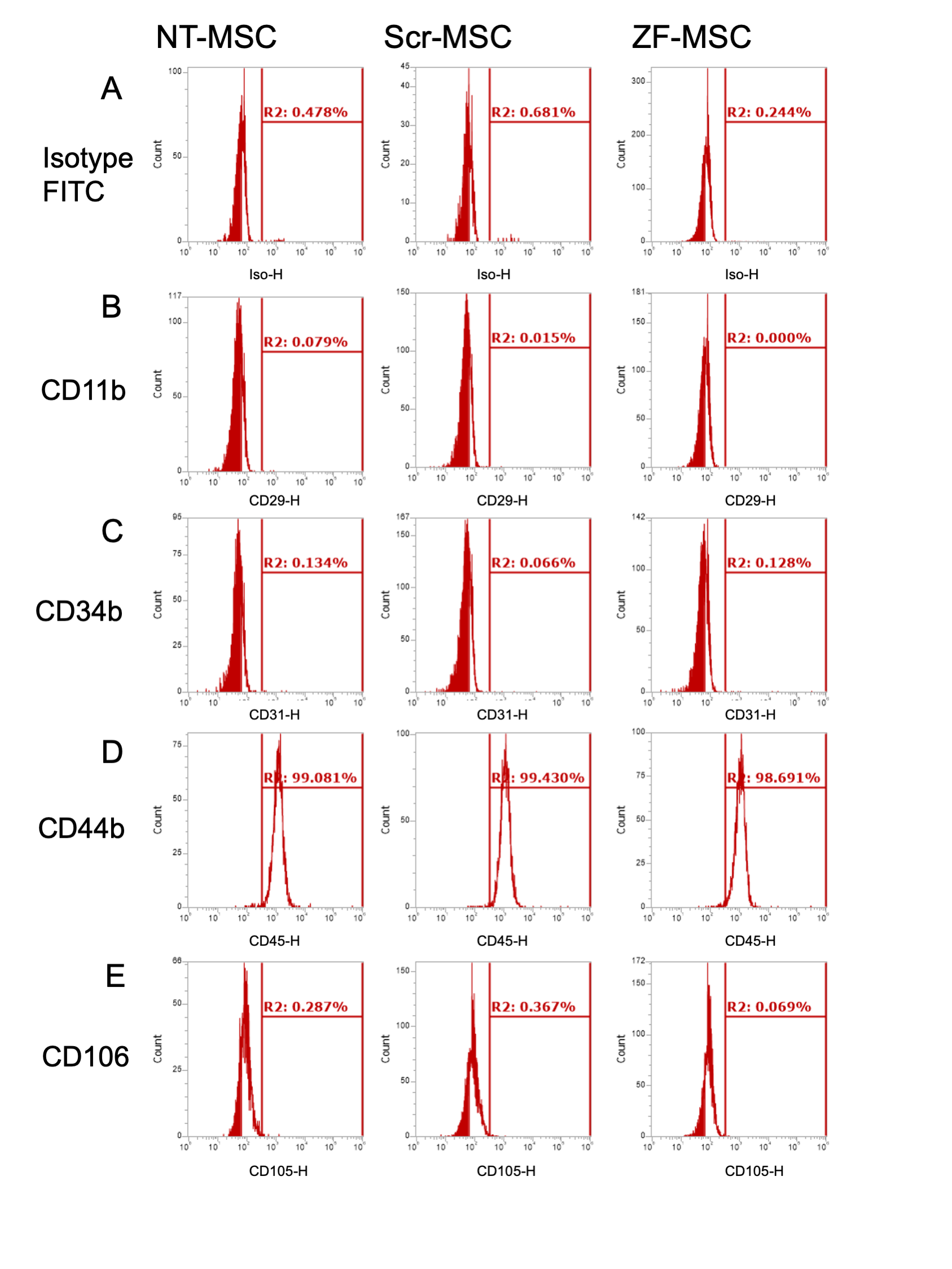

Supplement: Supplementary Figure 2 — Transduced mouse BM-MSCs demonstrate positive canonical MSC markers CD44b and negative for CD11b, CD34b, and CD106, related to Figure 1. (A) Isotype FITC negative control. (B) NT MSC, Scr-MSC, and ZF-MSCs are negative for immune marker CD11b. (C) NT MSC, Scr-MSC, and ZF-MSCs are negative for hemopoietic stem cell marker CD34b. (D) NT MSC, Scr-MSC, and ZF-MSCs are positive for MSC surface marker CD44b. (E) NT MSC, Scr-MSC, and ZF-MSCs are negative for vascular adhesion marker CD106. [file Image_2.TIFF]

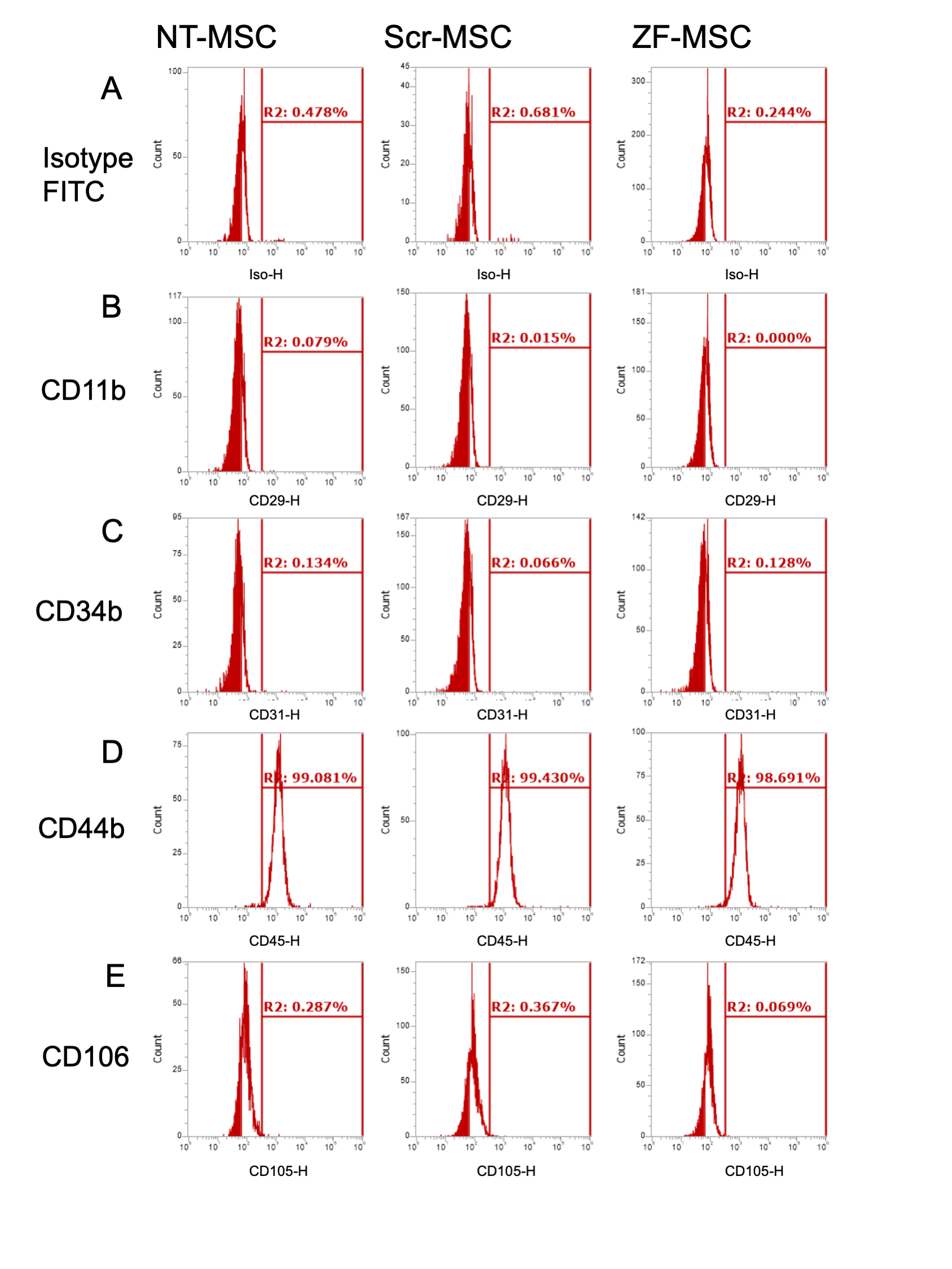

Supplement: Supplementary Figure 3 — Transduced mouse BM-MSCs demonstrate positive canonical MSC markers CD29b and Sca1 and negative for CD31, CD45, and CD105, related to Figure 1. (A) Isotype PE negative control. (B) NT MSC, Scr-MSC, and ZF-MSCs are positive for MSC surface marker CD29b. (C) NT MSC, Scr-MSC, and ZF-MSCs are negative for platelet endothelial adhesion molecular marker CD31. (D) NT MSC, Scr-MSC, and ZF-MSCs are negative for differentiated hemopoietic cell marker CD45b. (E) NT MSC, Scr-MSC, and ZF-MSCs are negative for vascular endothelial marker CD105. (F) NT MSC, Scr-MSC, and ZF-MSCs are enriched for mouse MSC marker Sca-1. [file Image_3.TIFF]

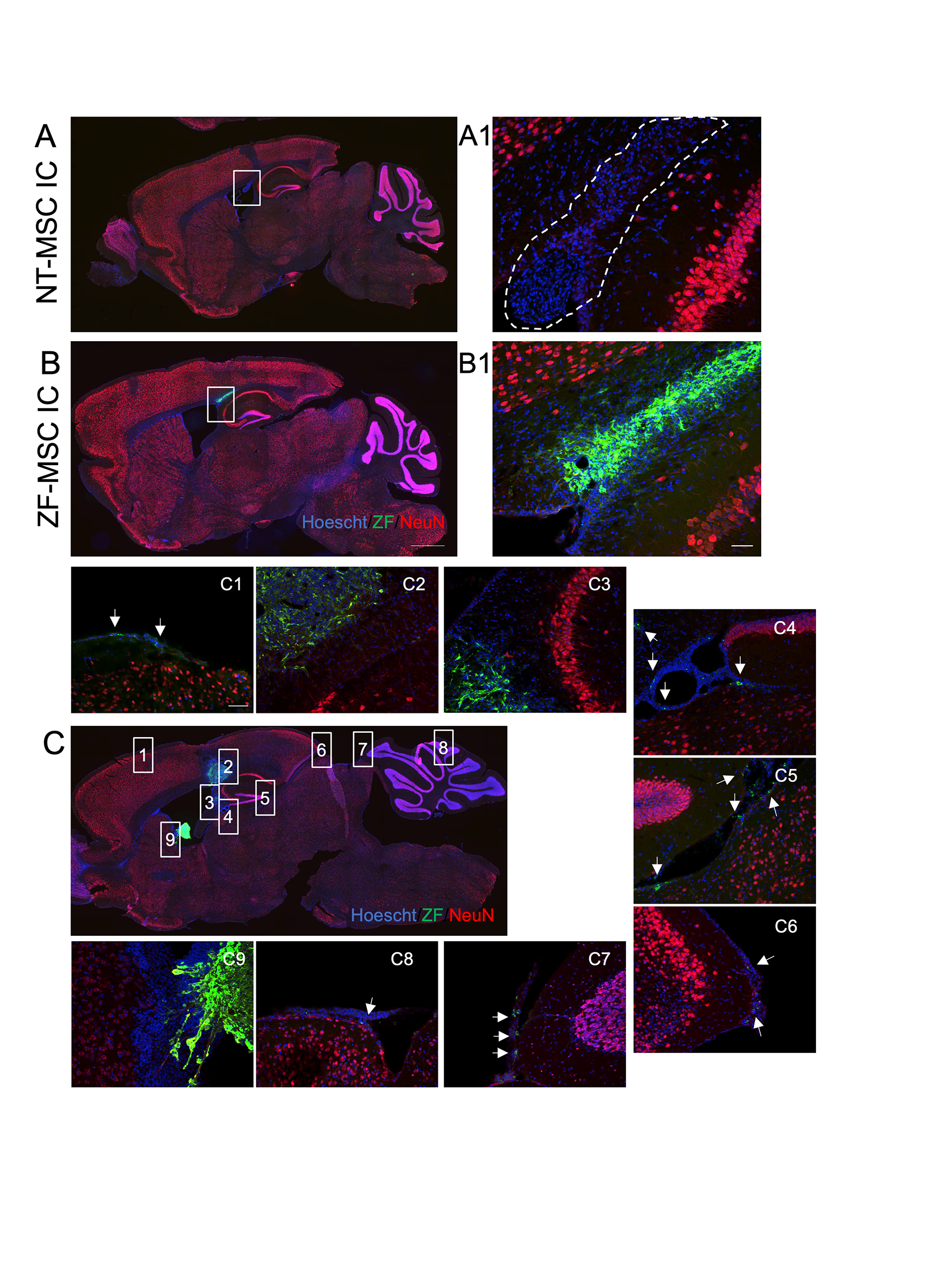

Supplement: Supplementary Figure 4 — Intracranial injection of ZF-MSC demonstrated motility from the parenchyma, related to Figure 4. (A) NT-MSC IC was observable within the mouse brain one week following injection and detected moving towards the lateral ventricles along the corpus callosum (A1). The white box denotes inlet of an image in panel (A1). Dashed white lines indicate the presence of injected MSC due to distinct morphology in contrast to mature NeuN+ (red) neurons and nuclear Hoechst (blue) labeling. (B) ZF-MSC IC (green) was observable within the mouse brain following injection and detected moving along the corpus callosum towards the lateral ventricles (B1). A white box denotes an inlet of image in panel (B1). (C) ZF-MSCs IC were detectable across multiple regions of the brain, including the forebrain (C1), site of injection along the corpus callosum (C2), anterior to the hippocampus (C3), along the third ventricle–ventral (C4) and posterior (C5) to the hippocampus, in the visual cortex (C6), along with the cerebellum (C7,C8), and within the lateral ventricles (C9). Scale bar = 500 μm (A–C), 50 μm (A1,B1,C1–C9). [file Image_4.TIFF]

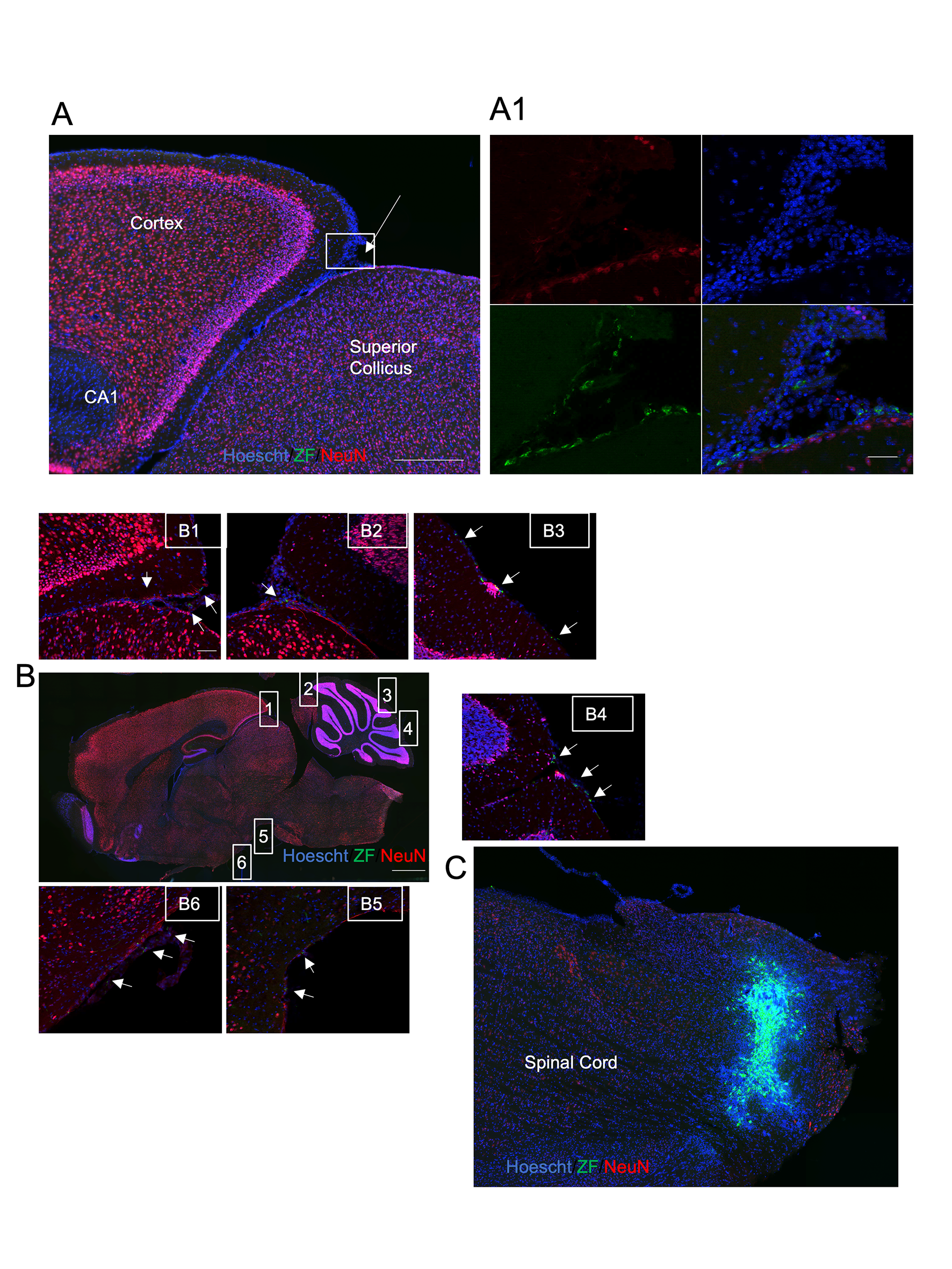

Supplement: Supplementary Figure 5 — Cisterna magna injection of ZF-MSC demonstrated motility to the CSF one week following injection, related to Figure 4. (A) Cisterna magna (CM) injected ZF+ (green) were detectable along the somatosensory cortex (A1) and were distinct from surrounding NeuN+ (red) neurons and other Hoechst (blue) labeled cells. (B) ZF-MSC injected via the CM were detectable across multiple regions including the cortex (B1), cerebellum (B2–B4) and along hypothalamic regions (B5–B6). (C) ZF-MSC injected via the CM were detectable within the mouse spinal cord. Scale bar = 500 μm (A–C), 50 μm (A1,B–B6). [file Image_5.TIFF]

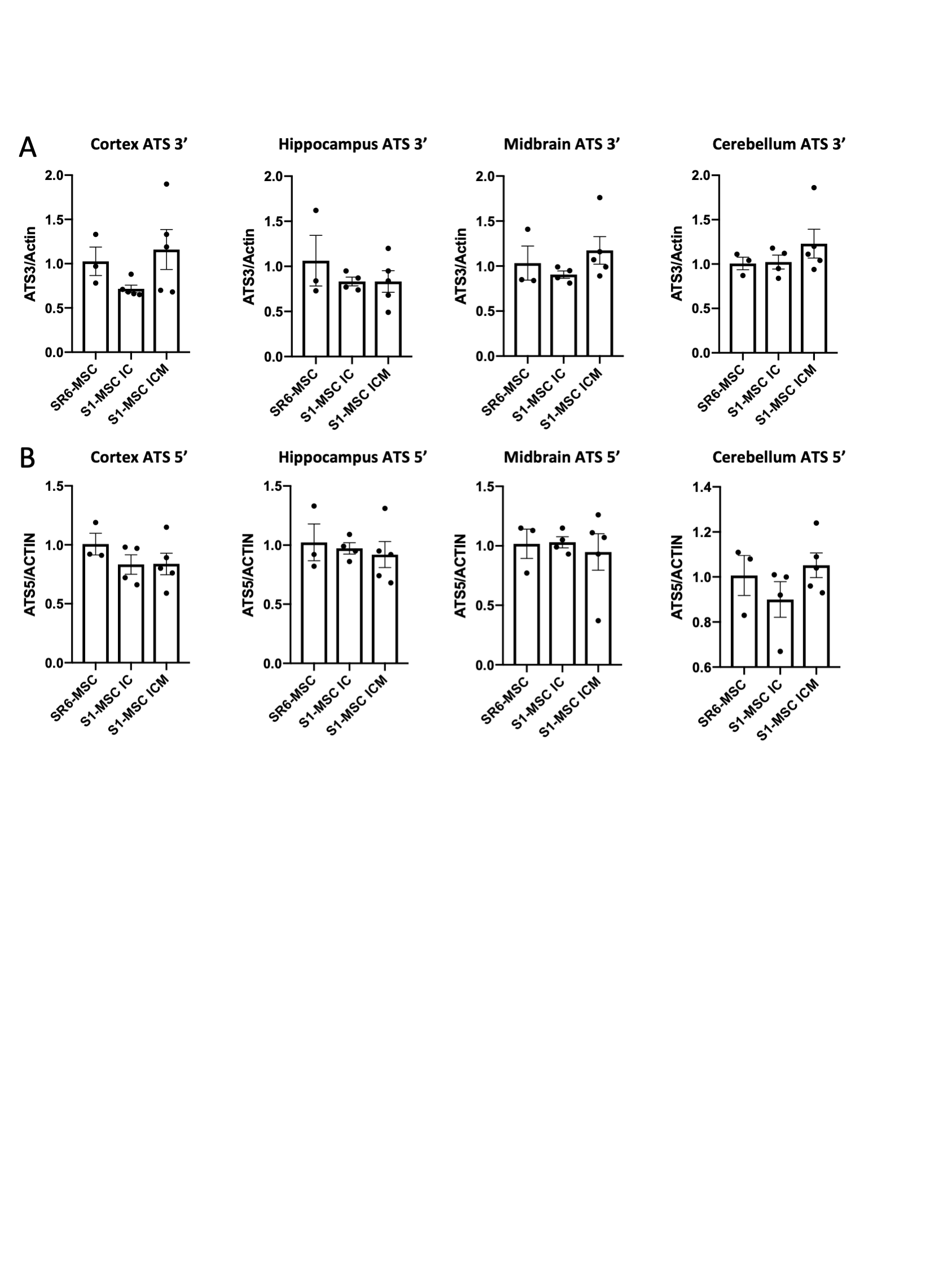

Supplement: Supplementary Figure 6 — YFP transcript expression is not altered with response to ZF, related to Figure 5. (A) No observable changes in the 3′ Ube3a-Ats transcript was observed in the cortex, hippocampus, midbrain, or cerebellum in 3-weeks post ZF-MSC treatment in Ube3a+/yfp mice. (B) No observable changes in the 5′ Ube3a-Ats transcript was observed in the cortex, hippocampus, midbrain, or cerebellum in 3-weeks post ZF-MSC treatment in Ube3a+/yfp mice. Error bars indicate ± standard error of the mean. Dots indicate individual mice. [file Image_6.TIFF]
