# Supplementary material for: An in vivo Cell-Based Delivery Platform for Zinc Finger Artificial Transcription Factors in Pre-clinical Animal Models
Source: Front Mol Neurosci. 2022 Jan 27;14:789913. doi: 10.3389/fnmol.2021.789913 (PMC8829036; doi:10.3389/fnmol.2021.789913)
Supplement: Supplementary file 7 [file Table_1.pdf]

Supplementary table 1

Table 1. mMSC Characterization Antibodies

MSC Characterization Antibodies	Vendor	Catalog
Rat IgG2 kappa isotype control, PE	eBioscience	12-4321-41
CD45 antibody, PE	eBioscience	12-0451-81
CD105 antibody, PE	eBioscience	12-1051-81
CD29 antibody, PE	eBioscience	12-0291-81
Sca1 antibody, PE	eBioscience	12-5981-81
Rat IgG2 kappa isotype control, FITC	eBioscience	11-4321-81
CD11b antibody, FITC	eBioscience	11-0112-81
CD31 antibody	BD Biosciences	550274
IgG Anti-rat Alexa Fluor 594	ThermoFisher	A11007
CD34 antibody, FITC	eBioscience	11-0341-81
CD44 antibody, FITC	eBioscience	11-0441-81
CD73 antibody, FITC	eBioscience	12-0731-81
CD106 antibody, FITC	eBioscience	11-1061-81
